# Cell-cycle inhibition and immune microenvironment in breast cancer treated with ribociclib and letrozole or chemotherapy

**DOI:** 10.1038/s41523-024-00625-7

**Published:** 2024-03-06

**Authors:** Tomás Pascual, Aranzazu Fernandez-Martinez, Yash Agrawal, Adam D. Pfefferle, Nuria Chic, Fara Brasó-Maristany, Blanca Gonzàlez-Farré, Laia Paré, Guillermo Villacampa, Cristina Saura, Cristina Hernando, Montserrat Muñoz, Patricia Galván, Xavier Gonzàlez-Farré, Mafalda Oliveira, Miguel Gil-Gil, Eva Ciruelos, Patricia Villagrasa, Joaquín Gavilá, Aleix Prat, Charles M. Perou

**Affiliations:** 1 Department of Genetics, Lineberger Comprehensive Cancer Center, University of North Carolina, Chapel Hill, NC USA; 2 SOLTI Cancer Research Group, Barcelona, Spain; 3 Medical Oncology Department, Hospital Clinic de Barcelona, Barcelona, Spain; 4 Translational Genomics and Targeted Therapies in Solid Tumors, August Pi i Sunyer Biomedical Research Institute (IDIBAPS), Barcelona, Spain; 5 Pathology Department, Hospital Clinic of Barcelona, Barcelona, Spain; 6 Medical Oncology Department, Vall d’Hebron Institute of Oncology (VHIO), Hospital Universitari Vall d’Hebron, Vall d’Hebron Barcelona Hospital Campus, Barcelona, Spain; 7 Breast Cancer Program, Vall d’Hebron Institute of Oncology (VHIO), Hospital Universitari Vall d’Hebron, Vall d’Hebron Barcelona Hospital Campus, Barcelona, Spain; 8 Medical Oncology Department, Hospital Clínico Universitario de Valencia, Valencia, Spain; 9 Breast Cancer Biology Research Group, Biomedical Research Institute INCLIVA, Valencia, Spain; 10 Breast Cancer Unit, Hospital Universitari General de Catalunya, Universitat Internacional de Catalunya, Barcelona, Spain; 11 IDIBELL, L’Hospitalet, Barcelona, Spain; 12 Department of Medical Oncology, Multidisciplinary Breast Cancer Unit, Institut Català d’Oncologia Medical Oncology, Barcelona, Spain; 13 Medical Oncology Department, Hospital 12 de Octubre, Madrid, Spain; 14 Medical Oncology Department, HM Hospitales Madrid, Madrid, Spain; 15 Fundación Instituto Valenciano de Oncología, Valencia, Spain; 16 Department of Medicine, University of Barcelona, Barcelona, Spain; 17 Breast Cancer Unit, IOB-Quirón Salud, Barcelona, Spain

**Keywords:** Breast cancer, Transcriptomics, Translational research, Tumour immunology

## Abstract

In this study, we performed genomic analyses of cell cycle and tumor microenvironment changes during and after ribociclib and letrozole or chemotherapy in the CORALLEEN trial. 106 women with untreated PAM50-defined Luminal B early breast cancers were randomly assigned to receive neoadjuvant ribociclib and letrozole or standard-of-care chemotherapy. Ki67 immunohistochemistry, tumor-infiltrating lymphocytes quantification, and RNA sequencing were obtained from tissue biopsies pre-treatment, on day 14 of treatment, and tumor specimens from surgical resection. Results showed that at surgery, Ki67 and the PAM50 proliferation scores were lower after ribociclib compared to chemotherapy. However, consistent reactivation of tumor cell proliferation from day 14 to surgery was only observed in the ribociclib arm. In tumors with complete cell cycle arrest (CCCA) at surgery, PAM50 proliferation scores were lower in the ribociclib arm compared to chemotherapy (*p* < 0.001), whereas the opposite was observed with tumor cellularity (*p* = 0.002). Gene expression signatures (GES) associated with antigen-presenting cells (APCs) and innate immune system activity showed increased expression post-chemotherapy but decreased expression post-ribociclib. Interferon-associated GES had decreased expression with CCCA and increased expression with non-CCCA. Our findings suggest that while both treatment strategies decreased proliferation, the depth and the patterns over time differed by treatment arm. Immunologically, ribociclib was associated with downregulated GES associated with APCs and the innate immune system in Luminal B tumors, contrary to existing preclinical data. Further studies are needed to understand the effect of CDK4/6 inhibition on the tumor cells and microenvironment, an effect which may vary according to tumor subtypes.

## Introduction

Hormone receptor-positive/HER2-negative (HR+/HER2−) breast cancer is clinically and biologically heterogeneous. At the gene expression level, the PAM50 assay^1,2^ has identified and intensively studied up to 4 intrinsic subtypes within HR+/HER2− breast cancer (i.e., Luminal A, Luminal B, HER2-enriched and Basal-like^3–7^. Compared to the PAM50 Luminal A subtype, the PAM50 Luminal B subtype is characterized by higher expression of proliferation/cell cycle-related genes, lower expression of several luminal-related genes such as the *PGR* and *FOXA1*^8^, and worse survival outcomes at 5- and 10-years irrespective of adjuvant systemic therapy^9–11^. To date, endocrine and cytotoxic therapies remain the standard of care for most patients with Luminal B disease^12,13^.

New targeted drugs have recently been incorporated to treat HR+/HER2− breast cancer. Among them, ribociclib, a CDK4/6 inhibitor, in combination with endocrine therapy (ET), has been shown to improve progression-free survival^14–16^ and overall survival^17–19^ over single agent ET in patients with metastatic HR+/HER2− breast cancer, including Luminal B disease^20,21^.

CDK4/6 inhibitors are traditionally combined with ET for improved efficacy in the neoadjuvant setting as well. A randomized window-of-opportunity trial demonstrated that ribociclib and letrozole combination therapy increased likelihood of complete cell cycle arrest (CCCA) in postmenopausal women with surgically resectable grade II/III HR+/HER2− breast cancer^22^. Another phase II trial, PALLET, showed that adding palbociclib to letrozole increased CCCA at 14 weeks in postmenopausal women with operable HR+/HER2− tumors^23^. Several studies, including NeoPalAna^24^, neoMONARCH^25^, and FELINE^26^, supported the efficacy of different CDK4/6 inhibitors combined with ET for achieving CCCA in various subsets of breast cancer patients. Furthermore, two studies (NeoPAL^27^ and CORALLEEN^28^) demonstrated comparable efficacy and superior safety of neoadjuvant CDK4/6 inhibitors + ET for high-risk luminal breast cancer compared to conventional neoadjuvant chemotherapy regimens.

In the CORALLEEN phase II trial, 6 months of neoadjuvant ribociclib in combination with ET showed high activity similar to neoadjuvant multi-agent chemotherapy but with better associated quality of life^29^ in 106 patients with Luminal B early breast cancer^28^. A large phase III clinical trial (NATALEE; NCT03701334) is evaluating 3 years of adjuvant ribociclib in combination with ET versus ET alone, for which interim results show improvement in disease-free survival with a well-tolerated side effect profile^30^.

Although CDK4/6 inhibitors have had great value in the treatment of metastatic HR+/HER2− breast cancer, a better understanding of their value is needed in early stage HR+/HER2− breast cancer for the following reasons. First, there is conflicting data for CDK4/6 inhibitor use in early stage breast cancer—two large phase III trials with adjuvant palbociclib (i.e., PALLAS^31^ and PENELOPE-B^32^) reported negative results, while the phase III trials of adjuvant abemaciclib and ribociclib (i.e., MONARCHE^33^ and NATALEE^30^) were positive. Second, although preclinical data has shown that CDK4/6 inhibitors can stimulate immune-related effects^34,35^, this has not been well-studied in patients or within different tumor intrinsic subtypes, and the early disease setting represents the perfect context to evaluate this phenomenon. Third, a more thorough understanding of the biological effects induced by CDK4/6 inhibitors might help to better select patients and optimize treatment decisions while, at the same time, to explore new treatment strategies such as using CDK4/6 inhibitors to replace (neo)adjuvant chemotherapy instead of adding these drugs after chemotherapy in patients with high-risk disease.

Here, we report an extensive analysis of a high-risk ER+/HER2− cohort to understand the effects of neoadjuvant ribociclib and letrozole or multi-agent chemotherapy on the tumor cell cycle and tumor microenvironment by analyzing samples before, during, and after 6 months of therapy in patients with newly diagnosed PAM50 Luminal B breast cancer who participated in the SOLTI-1402 CORALLEEN phase II randomized trial.

## Results

The demographics and primary clinical endpoint of the patients treated in CORALLEEN have been previously published^28^. In summary, baseline patient characteristics were similar between both treatment arms: mean age was 64 years, mean tumor size was 3.8 cm, clinical node-positive disease represented 39.0%, and the mean Ki67 by immunohistochemistry (IHC) was 33.2%. Patient characteristics in the subset of 83 patients with all biomarkers assessed at baseline were similar to those of the whole study. The consort diagram and a breakdown of the number of samples available for each biomarker at each time point is shown in Supplementary Fig. 1.

### Cell-cycle changes by Ki67

**Fig. 1:**
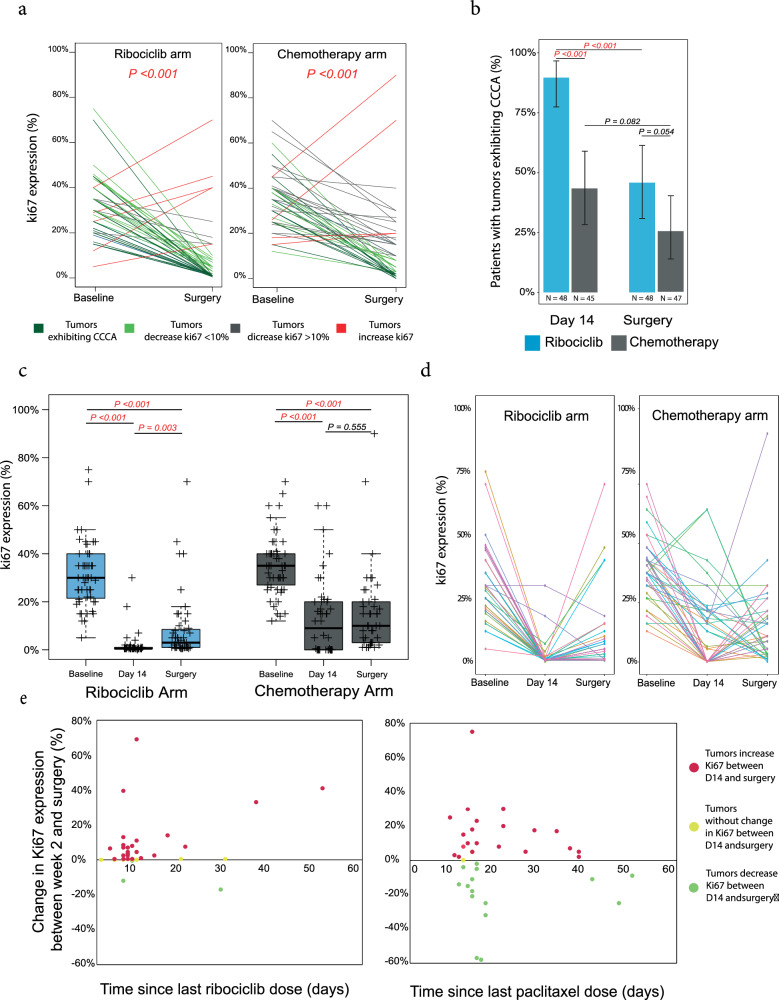
Comparative antiproliferative impact of ribociclib plus letrozole and multi-agent chemotherapy on luminal B Breast Cancer, as measured by Ki67 expression. **a** Individual paired Ki67 expression at baseline and surgery after treatment with ribociclib and letrozole and multi-agent chemotherapy. Colored lines represent individual patient data. **b** Mean percentage rate of response as determined by complete cell-cycle arrest (CCCA, Ki67 < 2.7%), at week 2 and surgery by treatment arms. **c** Expression of Ki67 across the three timepoints (Baseline, day 14, surgery) in ribociclib and chemotherapy arms. *p*-value was obtained after performing ANOVA test. **d** Individual paired Ki67 expression at baseline, day 14, and surgery. Colored lines represent individual patient data. **e** Change in Ki67 expression between week 2 and surgery by interval between ribociclib or paclitaxel discontinuation and surgery. Each point represents a patient.

The proportion of Ki67-positive tumor cells by IHC was assessed in 105 (99.1%), 93 (87.7%), and 95 (89.6%) tumor samples obtained at baseline, D14, and surgery, respectively (Supplementary Fig. 1B). The relative change in Ki67 between baseline and surgery was similar in both treatment arms (Fig. 1a), with a decrease in Ki67 observed in most samples at surgery. Between baseline and D14, Ki67 decreased with geometric mean change of −88.7% (95% CI, −82.3% to −92.7%) in the ribociclib plus letrozole arm, and by −79.3% (95% CI, −65.1% to −87.8%) in the chemotherapy arm. Between baseline and surgery, Ki67 expression decreased with a geometric mean change of −88.3% [95% confidence interval (95% CI, −82.1% to −92.3%) in the ribociclib plus letrozole arm, and −84.1% (95% CI, −70.5% to − 91.4%) in the chemotherapy arm.

The rate of CCCA at D14 was significantly higher in the ribociclib arm (43/48 patients [89.6%], 95% CI 77.3% to 96.5%) compared with the chemotherapy arm (19/45 patients [42.2%], 95% CI 27.7% to 57.8%) (*p* < 0.001). At the time of surgery, CCCA was observed in 22/48 patients (45.8%, 95% CI 31.4% to 60.8%) in the ribociclib arm, compared to 12/47 patients (25.5%, 95% CI 13.9% to 40.3%) in the chemotherapy arm (*p* = 0.054) (Fig. 1b).

Interestingly, in both the ribociclib and chemotherapy arms, there was a significant decrease in Ki67 expression between baseline and D14, and between baseline and surgery (*p* < 0.001); however, in the ribociclib arm there was a significant increase in Ki67 expression between D14 and surgery (*p* = 0.003) (Fig. 1c). Regarding Ki67 trends in each treatment arm, 73.3% and 57.5% of patients in the ribociclib and chemotherapy arms, respectively, demonstrated a relative increase in Ki67 expression between D14 and surgery (Fig. 1d and Supplementary Fig. 2). Proportionally more samples did so after ribociclib than after chemotherapy, and there is a weak correlation between time off ribociclib before surgery and increase in Ki67 at time of surgery from D14 (Fig. 1e).

A correlation between lower Ki67 levels in the surgical samples and treatment responses, assessed through magnetic resonance imaging (MRI), was observed in both treatment arms, as well between Ki67 levels and PAM50 risk-of-recurrence (PAM50-ROR) score^27^. (Supplementary Fig. 3). Within the chemotherapy arm, the MRI-based overall response rate (ORR) among patients with complete cell cycle arrest (CCCA) was 12 out of 13 compared to 25 out of 33 patients without CCCA (*p* = 0.207). In the ribociclib arm, patients with CCCA at the surgical stage exhibited an ORR of 17 out of 20, whereas patients without CCCA had an ORR of 11 out of 25 (*p* = 0.005). As anticipated, lower Ki67 levels correlated with PEPI score 0, but not with Residual Cancer Burden (RCB) at the time of surgery (Supplementary Figs. 3G–J).

### Tumor-specific gene expression changes at baseline by CCCA

To determine how gene expression was affected by neoadjuvant ribociclib and letrozole and by chemotherapy, we performed supervised gene expression analyses to compare gene expression data from baseline tumor biopsies to D14 and to surgical specimens. We transformed the gene expression data into a set of 660 previously published gene expression signatures representing many features of tumor cells and their microenvironment including >200 signatures of immune cells. The complete list of signatures used is shown in Supplementary Tables 1 and 2.

**Fig. 2:**
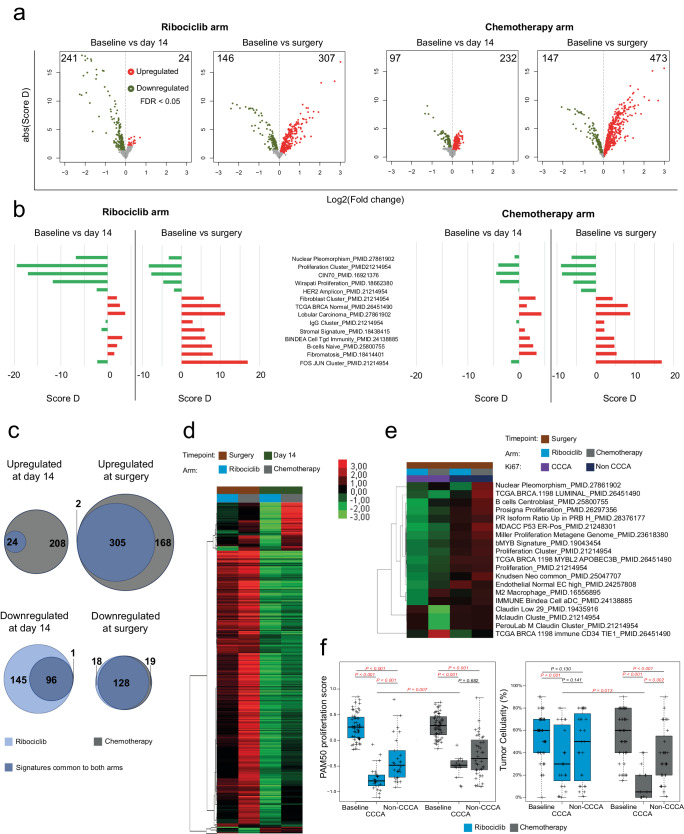
Dynamics of gene expression in Luminal B breast cancer: during and post-treatment with ribociclib plus letrozole versus multi-agent chemotherapy. **a** Volcano plots of log2 fold change of median gene expression and absolute Score D. Colors point to a significance threshold of FDR < 0.05. Significance was calculated using SAM paired samples analysis. **b** Bar plot of the D-score of selected signatures in ribociclib and chemotherapy arm between the 3 timepoints. **c** Venn diagram with signatures upregulated and downregulated (FDR < 0.05) between baseline and day 14 and between baseline and surgery in the ribociclib and chemotherapy arms. **d** Clusters with signatures selected from ratios between baseline and surgery according to timepoint and arm with a FDR < 0.01 in SAM multiclass analysis. **e** Clusters with signatures selected from ratios between baseline and surgery according to complete cycle arrest (CCCA) and arm with a FDR < 0.01 in SAM multiclass analysis. **f** PAM50 proliferation score and percentage of tumor cellularity at baseline and surgery according to CCCA or non- CCCA at surgery. *p*-value was obtained after performing ANOVA test.

Paired two-class SAM analysis^36^ comparing D14 samples to baseline showed 24 (3.6%) and 241 (36.5%) signatures with increased and decreased expression in the ribociclib arm, and 232 (35.2%) and 97 (14.7%) signatures with increased and decreased expression in the chemotherapy arm (FDR < 5%). Comparing post-ribociclib surgical specimens to baseline biopsy specimens identified 307 (46.5%) signatures with increased expression and 146 (22.1%) with decreased expression (FDR < 5%). Post-chemotherapy surgical samples had 473 (71.6%) and 147 (22.3%) signatures with increased and decreased expression, respectively (Fig. 2a).

Common to two arms, at both D14 and surgery, decreased expression of proliferation-related signatures and an increased expression of signatures associated with normal breast stroma were observed (Fig. 2b). At day 14, 232 signatures were upregulated following a single dose of anthracycline-based chemotherapy, whereas only 24 signatures showed significant upregulation in the ribociclib arm, all of which were also upregulated after chemotherapy. On the other hand, 241 signatures were downregulated in the ribociclib arm, and 97 were downregulated chemotherapy arm, of which 96 were shared with the ribociclib arm (Fig. 2c). In the surgical samples, 305 signatures exhibited significantly increased expression common to both treatment arms, with 2 signatures unique to the ribociclib arm and 168 unique to the chemotherapy arm. Furthermore, 128 signatures showed significantly decreased expression in both arms, with 18 unique to the ribociclib arm and 19 unique to the chemotherapy arm (Fig. 2c).

Supervised hierarchical clustering was performed on D14 and surgical samples in both arms. The resulting heatmap of median signature expression is shown in Fig. 2d, which demonstrated a large cluster of signatures with decreased expression at D14 that subsequently showed increased expression in specimens at time of surgery. These signatures are primarily associated with normal breast stroma. A smaller cluster of signatures had increased expression in the chemotherapy arm but decreased expression in the ribociclib arm, particularly at D14; these were primarily associated with cell proliferation.

With respect to PAM50 subtype, most tumors demonstrated “subtype-switching” from Luminal B predominantly to Luminal A subtype, particularly with ribociclib. On D14, 48/52 (92.3%) of tumors in the ribociclib arm had switched to Luminal A subtype, and 19/54 (35.2%) of tumors in the chemotherapy arm had switched Luminal A subtype. At time of surgery, 44/52 (84.6%) of tumors in the ribociclib arm were Luminal A, and 43/54 (79.6%) of tumors in the chemotherapy arm were Luminal A. Of note, 3 patients’ tumors in each study arm had switched back to Luminal B subtype at time of surgery after switching to Luminal A at D14 (Supplementary Fig. 4).

Next, the surgical samples were stratified by CCCA status, and paired two-class SAM analysis identified 19 gene expression signatures differentially expressed between CCCA and non-CCCA samples. Supervised hierarchical clustering was performed on these samples. Signatures corresponding to proliferation had lower expression in tumors achieving CCCA than in non-CCCA tumors in both arms and had lower expression after ribociclib than after chemotherapy irrespective of CCCA status. Post-chemotherapy CCCA samples also showed markedly lower expression of claudin-low-related signatures (Fig. 2e), thus becoming more claudin-low-like as has been demonstrated previously post-chemotherapy^37^.

PAM50 proliferation scores were significantly decreased after ribociclib and after chemotherapy between baseline and time of surgery for both CCCA and non-CCCA samples (*p* < 0.001), which is consistent with the decreased expression seen of proliferation-related gene expression profiles (Fig. 2f). Of note, PAM50 proliferation scores were also significantly lower in the setting of CCCA after ribociclib than after chemotherapy (*p* = 0.007), and in the ribociclib arm were lower with CCCA samples than non- CCCA samples (*p* < 0.001), while there was no significant difference in PAM50 proliferation scores between CCCA and non-CCCA samples in the chemotherapy arm (*p* = 0.682). Additionally, while CCCA samples were associated with a pronounced decrease in tumor cellularity from baseline in both arms (*p* < 0.001), cellularity was significantly further decreased post-chemotherapy than post-ribociclib (*p* = 0.013). Post-chemotherapy cellularity was significantly lower in CCCA samples than non-CCCA samples (*p* = 0.002), while this was not the case post-ribociclib (*p* = 0.141) (Fig. 2f). This speaks to the differing mechanisms by which ribociclib and AC-T chemotherapy achieve CCCA, with the former brought about by targeted inhibition to induce Rb-dependent cell cycle arrest, while AC-T induces cytotoxicity through interference with DNA duplication and microtubule formation.

### TILs changes

Stromal TILs were measured in 104 (98.1%), 97 (91.5%) and 93 (87.7%) tumor samples obtained at baseline, D14 and surgery, respectively. Most samples (83.9%) had ≤10% TILs; 5 (4.7%) showed lymphocyte-predominant breast cancer (≥50% TIL^38,39^). TIL quantification observed post-treatment at time of surgery was not significantly different in either arm compared to baseline (ribociclib arm: *p* = 0.161; chemotherapy arm: *p* = 0.830) (Supplementary Fig. 5A); this was also the case between baseline and D14, and between D14 and surgery (Supplementary Fig. 5B, D). Stratified by CCCA status, the only significant difference between subgroups was observed in the chemotherapy arm, with greater TILs observed in CCCA samples than non-CCCA samples (*p* = 0.017), though neither were significantly changed compared to baseline. There was no significant difference in TILs between CCCA and non-CCCA samples in the ribociclib arm, nor compared to baseline (Supplementary Fig. 5C). Of note, TIL quantification correlated with T-cell-associated gene expression signatures, though this correlation tended to be moderate (i.e., correlation coefficients ~ 0.5, see Supplementary Fig. 6).

### Immune gene expression changes

**Fig. 3:**
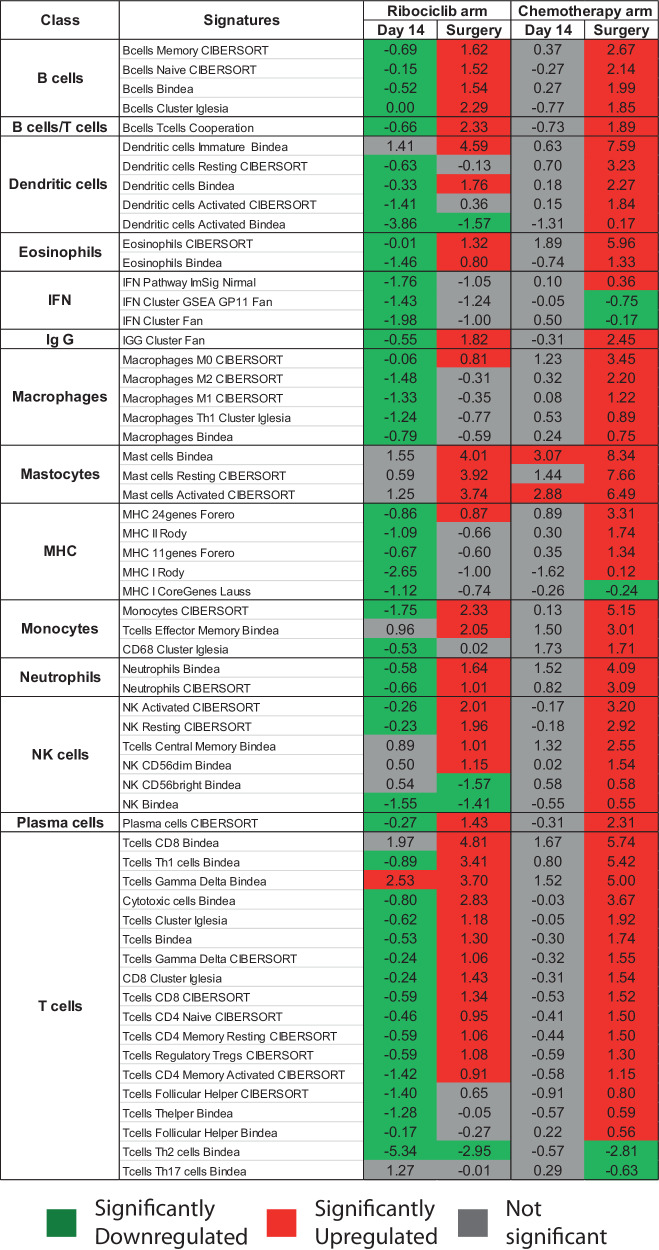
Comparative analysis of immune signature expression through supervised gene expression analysis between day 14 samples and surgery tumor specimens, categorized by different treatment arms. The numerical values correspond to Fold Change, computed using a two-class paired Significance Analysis of Microarrays (SAM), highlighting alterations. Color-coded boxes identify modules exhibiting significant changes with a false discovery rate (FDR) of ≤ 0.05, with upregulation in red and downregulation in green.

Supervised gene expression analysis identified significant expression changes in immune gene expression profiles in both arms at D14 and at surgery. At D14, ribociclib was associated with a significant decrease in gene expression signatures associated with most immune cell types. However, in both ribociclib and chemotherapy arms, at the time of surgery gene expression signatures generally showed a trend towards significantly increased expression across immune signature classes, though differences are present with nonsignificant decreases in expression associated with interferon signatures, macrophage signatures, and MHC signatures post-ribociclib (Fig. 3).

**Fig. 4:**
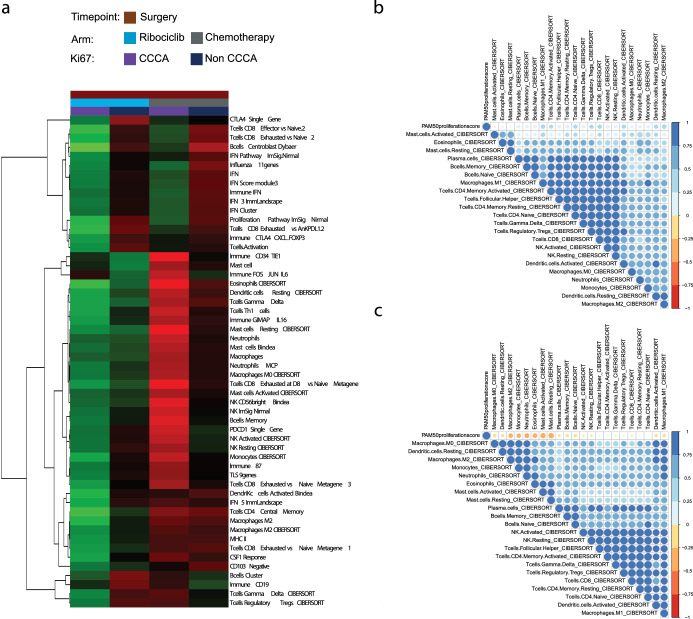
Differential immune gene expression signatures and correlations in Luminal B breast cancer surgical samples post neoadjuvant treatment. **a** Expression of selected signature in surgical samples with complete cycle arrest (CCCA) and non-CCCA in ribociclib arm (left) and chemotherapy arm (right). **b** Spearman correlation matrix for continuous PAM50 proliferation score and cibersort immune population in surgical samples after neoadjuvant ribociclib and letrozole. **c** Spearman correlation matrix for continuous PAM50 proliferation score and cibersort immune population in surgical samples after neoadjuvant multi-agent chemotherapy.

Next, we investigated how immune populations correlated with CCCA status after neoadjuvant treatment in both arms (i.e., at surgery specimens). A marked difference in immune signature expression emerged between the two treatment groups, stratified based on CCCA and non-CCCA statuses. Supervised gene expression analysis identified differentially expressed signatures between these groups. As seen in Fig. 4a, for significant immune signatures with an FDR < 0.01, there was almost universally decreased expression among tumors in the ribociclib arm that achieved CCCA. CCCA tumors in both arms showed reduced expression of interferon signatures, though this was more pronounced in the ribociclib arm. In post-treatment chemotherapy arm specimens, particularly for tumors achieving CCCA, there was increased expression of signatures associated with components of innate immunity, such as NK cells and neutrophils, as well as antigen-presenting cells, such as macrophages, monocytes, and dendritic cells; these signatures conversely showed decreased expression in post-treatment ribociclib arm specimens. In a broader context, we observed a conspicuous augmentation in immune infiltration in post-ribociclib samples with a higher tumor cell proliferation, in contrast to post-chemotherapy samples with lower proliferation. (Fig. 4b, c).

## Discussion

The SOLTI-1402 CORALEEN phase II study results from 2020 demonstrated molecular downstaging of high-risk early stage Luminal B tumors among patients treated with neoadjuvant letrozole and ribociclib as shown by high degree of intrinsic subtype conversion to Luminal A, decrease in Ki67 immunohistochemistry expression, and high proportion of patients with low risk of recurrence disease at time of surgery, as defined by their PAM50 risk-of-recurrence (PAM50-ROR) scores (<=40 if node-negative, <=15 if node-positive with 1–3 positive lymph nodes)^28^. In this study, we aimed to utilize molecular analysis of tissue samples from these patients to better understand the effects of therapy in each arm on cell-cycle inhibition, gene expression, and the tumor immune microenvironment.

Though change in Ki67 was comparable between ribociclib plus letrozole and chemotherapy arms between baseline and surgery, there was a higher rate of CCCA with the former. Furthermore, while both tumor cellularity and PAM50 proliferation scores were decreased from baseline in both arms in CCCA samples, PAM50 proliferation scores were significantly lower in CCCA tumors post-ribociclib arm compared to chemotherapy arm, while the converse was true for tumor cellularity. These results speak to the differences in mechanism of achieving CCCA between the two treatments; while AC-T has a direct cytotoxic effect through DNA damage and anti-microtubule activity to induce cell death, ribociclib and letrozole exert their effects through inhibition of key cell cycle regulators to induce cell cycle arrest. This is further supported by the rebound in Ki67 expression between D14 and surgery in the ribociclib arm, which may relate to time off treatment and suggests reversibility in the effects of ribociclib and letrozole, which does not occur after AC-T. This rebound effect has been consistently seen in neoadjuvant studies with other CDK4/6 inhibitors, such as neoMONARCH^25^ and NeoPalAna^24^.

On a gene expression level, while both arms demonstrate a decrease in proliferation signatures and an increase in breast stromal signatures, supervised gene expression analysis identified a cluster of claudin-low signatures that was particular to CCCA tumors in the chemotherapy arm, but not non-CCCA tumors or tumors in the ribociclib arm, suggesting an enrichment in cells in these tumors with tumor-initiating properties post-chemotherapy. Interestingly, both traditional cytotoxic chemotherapy and letrozole monotherapy have been demonstrated to generate enrichment in cell populations with tumor-initiating and mesenchymal properties^37^; however, on a gene expression level, ribociclib and letrozole in combination do not appear to demonstrate such an effect in our study. In preclinical studies, CDK4/6 inhibition has been shown to decrease cancer stem cell (CSC) populations in breast cancer cell lines^40,41^, which might help explain why claudin-low gene expression signature enrichment is absent with CCCA in the ribociclib arm.

Most patients in this study had low TIL presence, and TIL quantification did not significantly change with treatment in either arm. Pre-treatment TILs have been shown to correlate with prognosis and with neoadjuvant chemotherapy response in HR+/HER2− breast cancer^42,43^. However, our findings suggest that changes in TIL quantification may not be helpful as a marker of response or as an all-encompassing indicator of immune microenvironment change in the Luminal B population. This is supported by data in the preclinical setting where CDK4/6 inhibitors have not been demonstrated to have effects on the fractions of most types of TILs, and although they have been associated with increased CD3+ cells and decreased Treg infiltration^35^, overall TIL fraction may not reflect these more nuanced changes in the immune microenvironment in Luminal B tumors.

Gene expression analysis also highlighted marked differences in the post-treatment tumor immune microenvironment between the treatment arms. Gene expression signatures associated with antigen-presenting cells had increased expression in CCCA tumors post-chemotherapy, while the same modules showed decreased expression after ribociclib and letrozole. On the other hand, interferon-associated signatures are associated with CCCA status in both arms, namely with reduced expression in CCCA tumors and increased expression in non-CCCA tumors. De Angelis et al.^44^ revealed an intriguing connection between resistance to CDK4/6 inhibitors and abnormal activation of IFN-signaling, as showcased in both HR+/HER2− breast cancer cell lines of the Luminal B phenotype^45^, as well as patient tumors from the NeoPalAna and neoMONARCH neoadjuvant clinical trials. Similarly, heightened IFN-signaling activity was observed in preclinical ER+/HER2− breast cancer models that had developed acquired resistance to palbociclib, indicating a possible role of this signaling pathway in driving resistance mechanisms.

One preclinical study investigating CDK4/6 inhibitors suggested these agents are associated with increased gene expression associated with type III interferons and antigen processing and presentation pathways^35^. This study also analyzed expression data from NeoPalAna, a single-arm phase II study that investigated neoadjuvant palbociclib for ER+ breast cancer^24^, and showed that compared to pre-treatment tumors, biopsies of tumors after 15 days as well as after 12 weeks of palbociclib had increased expression of GSEA signatures associated with inflammatory response and interferon-gamma response. The neoMONARCH study similarly demonstrated the upregulation of GSEA signatures associated with interferon-gamma response, antigen cross-presentation, and PD-1 signaling after 16 weeks of treatment^25^. Of note, although the former did show downregulation of cell cycle-associated GSEA signatures from NeoPalAna expression data, these studies did not investigate how CCCA status correlated with immune gene expression; this may merit further investigation, as our analysis did note a positive correlation between proliferation and immune population after ET and ribociclib. Additionally, our study is notably focused on Luminal B tumors alone, which complicates a direct comparison of results with studies like NeoPalAna where non-Luminal B subtypes comprised 62% of the tumors with available RNAseq expression data and 51% of patients who had available molecular subtype data.

Our study has several strengths. Most patients had data for Ki67, TILs quantification, PAM50 proliferation scores and RNASeq data across both arms and across the treatment courses, including pre-treatment, D14, and at time of surgery. With these data, we were able to conduct a unique correlative study between gene expression, the tumor microenvironment, and CCCA status with both CDK4/6 inhibition and standard-of-care AC-T neoadjuvant therapy. Our study helps shed further light on CDK4/6 inhibitor effects on the tumor immune microenvironment in the clinical setting.

Conversely, our study also carries limitations. This is a retrospective exploratory study; therefore, confounding effects cannot be wholly excluded from our analysis, including from comparisons between treatment arms. A limited sample size constrains our ability to detect statistically significant differences between the groups, particularly when performing subgroup analysis with respect to CCCA status. Furthermore, we were unable to correlate our findings with survival outcomes, as the CORALLEEN study itself was exploratory and not powered to formally compare PAM50-ROR-low patients between study arms, and long-term follow-up was not available. To overcome these issues, we are currently running the phase II clinical trial RIBOLARIS (NCT05296746), which has started accrual and will evaluate ribociclib with letrozole without chemotherapy in the neo/adjuvant setting for those patients who achieve a PAM50-ROR-low status.

In conclusion, our study sheds light on differences in how ribociclib and letrozole may affect tumor biology of Luminal B breast cancer compared to AC-T in the neoadjuvant setting. Both therapies predictably lead to a decrease in Ki67 and increase in CCCA in both arms, although likely through differing mechanisms as demonstrated on cellular and gene expression levels. Our study shows the downregulation of antigen-presenting cells and components of innate immunity with CDK4/6 inhibitor plus aromatase inhibitor treatment particularly for tumors achieving CCCA, in contrast with preclinical data suggesting the converse effect. Further studies are essential to better understand how CDK4/6 inhibitors affect the tumor microenvironment and how they might most effectively be incorporated into tailored treatment strategies for managing early stage HR+/HER2− breast cancer.

## Methods

### Coralleen study

The main results of the CORALLEEN neoadjuvant phase II study have been previously reported^28^. This study is registered with ClinicalTrials.gov, number NCT03248427, and it is completed. The CORALLEEN trial was conducted under Good Clinical Practice guidelines and the Declaration of Helsinki, and the study protocol was approved by independent ethic committee of Hospital Vall d’Hebron. All patients provided written informed consent.

Briefly, postmenopausal women aged 18 years or older were accrued in this prospective, multicentric, randomized, parallel, non-comparative phase II clinical trial if they had an HR+/HER2− stage I-IIIA breast tumor with primary tumor size of at least 2 cm in diameter by magnetic resonance imaging (MRI) and a Prosigna®-defined Luminal B intrinsic subtype).

A total of 106 eligible patients were randomized in a 1:1 ratio to (A) ribociclib plus letrozole, or (B) multi-agent chemotherapy. Randomization was stratified based on tumor size (T3 vs. T1/T2) and nodal involvement (yes vs. no). Patients randomized to arm A received 28-day cycles of continuous daily letrozole, 2.5 mg per day, and ribociclib, 600 mg per day, according to a 3 weeks on/1 week off schedule, for a total duration of 24 weeks. Dose modifications were allowed to manage grade 2 or higher non-hematological adverse events and grade 3–4 hematological events. Two levels of dose reduction for ribociclib were prespecified: 400 mg/day on the first reduction and 200 mg/day on the second reduction. Patients discontinuing ribociclib treatment due to treatment-related toxicity could continue the active treatment phase of the study, receiving letrozole monotherapy as per the investigator’s discretion. Patients randomized to the standard chemotherapy arm received four cycles of doxorubicin 60 mg/m^2^, cyclophosphamide 600 mg/m2 administrated intravenously every 21 days, followed by weekly paclitaxel 80 mg/m2 intravenously for 12 weeks (AC-T). Surgery was done within 7 days after the last dose of ribociclib or 14 days after the last dose of chemotherapy. In the ribociclib plus letrozole group, letrozole was continued until the day of surgery. Tumor samples were collected according to protocol at baseline, day 14, and surgery, and subsequently formalin-fixed paraffin-embedded (FFPE).

### Pathology review

Hematoxylin and Eosin (H&E) stained sections from each sample were subjected to an independent central pathology review. At baseline, an H&E section was examined to confirm the presence of invasive tumor cells and to determine the minimum tumor surface area. For samples obtained on day 14 (D14) and surgery, those without invasive tumor cells were also profiled.

An immunohistochemical (IHC) study for Ki67 was carried out with a mouse monoclonal primary antibody (clone MIB-1) reactive in FFPE tissue sections using a peroxidase-labeled detection system, standard antigen retrieved protocols and BenchMark Ultra autostainer IHQ/ISH system Roche. Samples were blindly assessed by a pathologist using standard scoring guidelines^46^. CCCA was defined as Ki67 values < 2.7%^23–25,47^.

Stromal Tumor-infiltrating lymphocytes (TILs) at baseline, D14, and surgery were centrally evaluated on whole sections of tumor tissue stained with H&E blinded from clinical-pathological and outcome data. Percentages of TILs at baseline and D14 were scored in slides of core biopsies. TILs were quantified according to the Guidelines developed by the International TILs Working Group^38,39^. The reproducibility of this method has been described previously^48^.

### Gene expression profiling nCounter

For RNA purification (High Pure FFPET RNA isolation kit, Roche, Indianapolis, IN, USA), at least 1–5 10 μm FFPE slides were used for each tumor specimen, and macrodissection was performed (when needed) to avoid contamination with normal breast tissues. From each sample, 100 ng of RNA was hybridized to the human nCounter Breast Cancer 360 (BC360) gene expression panel^49,50^ (NanoString Technologies) and processed on the nCounter (NanoString Technologies) according to the manufacturer’s protocols. The reporter-code- count files generated for each sample were forwarded to NanoString Technologies for analysis. The raw count data were log2-transformed and normalized to housekeeping genes. These data were used to calculate the PAM50 subtype calls for each sample using proprietary algorithms.

### RNA sequencing, gene expression data values and normalization

Gene expression profiles were generated by RNA sequencing using an Illumina NovaSeq6000 and a rRNA-depletion method. Briefly, 100–1000 ng total RNA was converted to RNA sequencing (RNAseq) libraries using the TruSeq Stranded Total RNA Library Prep Kit with RiboZero Gold (Illumina) and sequenced on an Illumina NovaSeq 6000 using a 2 × 50 bp configuration. Quality-control-passed reads were aligned to the human reference CGRh38/hg38 genome using STAR^51^. Transcript abundance estimates for each sample were performed using Salmon^52^, an expectation-maximization algorithm using the UCSC gene definitions. Raw read counts for all RNAseq samples were normalized to a fixed upper quartile^53^. The raw read files are available in EGA (accession EGAD00001010121). The complete list of signatures used is shown in Supplementary Tables 1, 2.

### Gene expression signatures

We applied a collection of 660 gene expression modules, representing multiple biological pathways and cell types, to all tumor samples. These signatures were obtained from 108 publications partially summarized previously^54–56^ and 42 Gene set enrichment analysis (GSEA) signatures published in the Molecular Signature Database^57^. In detail, the 660 modules were calculated as the median value of each gene expression value present in the signature for each sample of the set used.

### Statistical analysis

To identify genes and signatures whose expression was significantly different between paired samples (baseline vs. D14 or baseline vs. surgery), we used a two-class paired Significance Analysis of Microarrays (SAM) with a false discovery rate (FDR) < 5%. All statistical tests were two-sided, and the statistical significance level was set to less than 0.05. We used R version 4.2.2 for all the statistical analyses.

### Supplementary information

SUPPLEMENTAL MATERIAL

reporting-summary

## Data Availability

The raw read files are available in EGA (accession EGAD00001010121). The datasets used and/or analyzed during the current study are available from the corresponding author on reasonable request.

## Code availability

All analyses were performed with R version 4.2.2 and Microsoft Excel. R codes are available from the corresponding author on reasonable request.
