# Supplementary material for: Cell-cycle inhibition and immune microenvironment in breast cancer treated with ribociclib and letrozole or chemotherapy
Source: NPJ Breast Cancer. 2024 Mar 6;10:20. doi: 10.1038/s41523-024-00625-7 (PMC10918094; doi:10.1038/s41523-024-00625-7)
Supplement: Supplementary file 1 — SUPPLEMENTAL MATERIAL [file 41523_2024_625_MOESM1_ESM.pdf]

Supplementary Figures

Supplementary Figure 1 ……………………………………………..1

Supplementary Figure 2 ……………………………………………..3

Supplementary Figure 3 ……………………………………………4

Supplementary Figure 4 ……………………………………………6

Supplementary Figure 5 ……………………………………………7

Supplementary Figure 6 ……………………………………………8

Supplementary Table 1 ……………………………………………9

Supplementary Table 2 …………………………………………..18

Supplementary Figure 1

A) Consort Diagram B) Diagram of the shared or individual tumor Ki67 IHC, TILs levels, PAM50 and RNA sequencing data successfully performed on each of the 106 patients of CORALLEEN trial.

A)

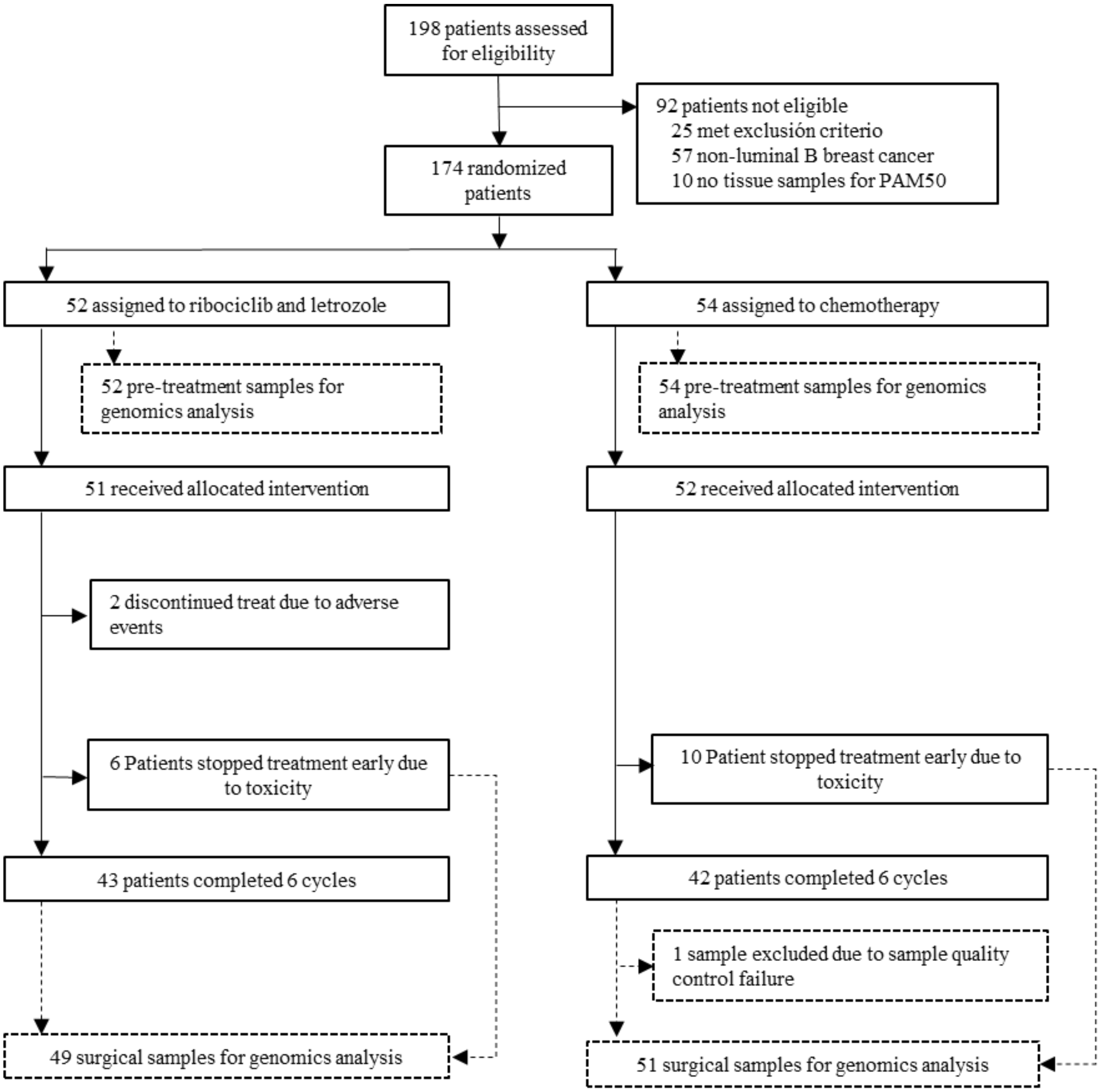

B)

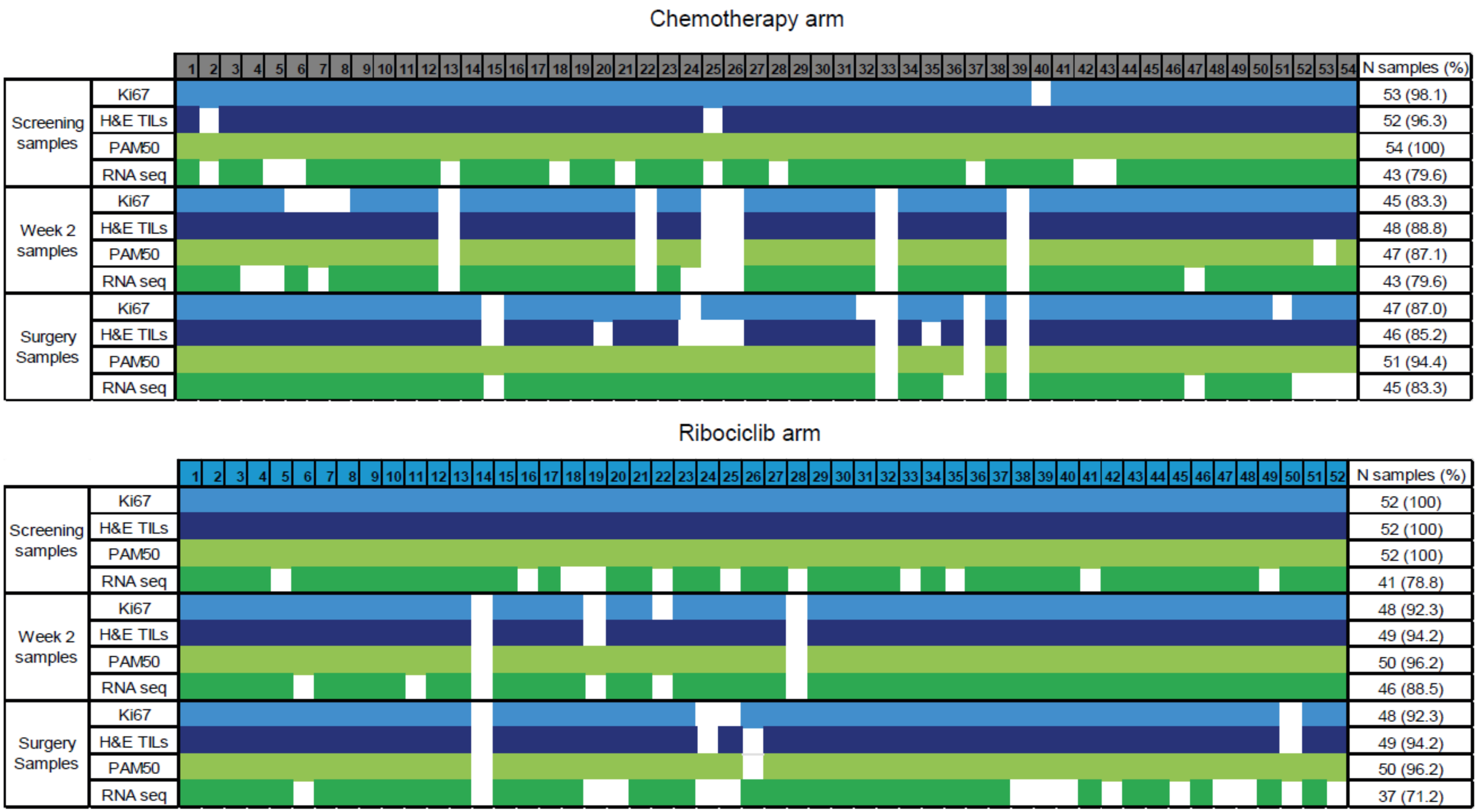
Chemotherapy arm
1 2 3 4 5 6 7 8 9 10 11 12 13 14 15 16 17 18 19 20 21 22 23 24 25 26 27 28 29 30 31 32 33 34 35 36 37 38 39 40 41 42 43 44 45 46 47 48 49 50 51 52 53 54
N samples (%)
Screening samples
Ki67
53 (98.1)
H&E TILs
52 (96.3)
PAM50
54 (100)
RNA seq
43 (79.6)
Week 2 samples
Ki67
45 (83.3)
H&E TILs
48 (88.8)
PAM50
47 (87.1)
RNA seq
43 (79.6)
Surgery Samples
Ki67
47 (87.0)
H&E TILs
46 (85.2)
PAM50
51 (94.4)
RNA seq
45 (83.3)
Ribociclib arm
1 2 3 4 5 6 7 8 9 10 11 12 13 14 15 16 17 18 19 20 21 22 23 24 25 26 27 28 29 30 31 32 33 34 35 36 37 38 39 40 41 42 43 44 45 46 47 48 49 50 51 52
N samples (%)
Screening samples
Ki67
52 (100)
H&E TILs
52 (100)
PAM50
52 (100)
RNA seq
41 (78.8)
Week 2 samples
Ki67
48 (92.3)
H&E TILs
49 (94.2)
PAM50
50 (96.2)
RNA seq
46 (88.5)
Surgery Samples
Ki67
48 (92.3)
H&E TILs
49 (94.2)
PAM50
50 (96.2)
RNA seq
37 (71.2)

Supplementary Figure 2

Changes in Ki67 levels across 3 time-points. (A) Ribociclib and letrozole arm; (B) Chemotherapy. Green line reflects any decrease in Ki67 levels between 2 time-points and red line reflects any increase in Ki67 levels. p-value was obtained after performing Wilcoxon signed-rank test.

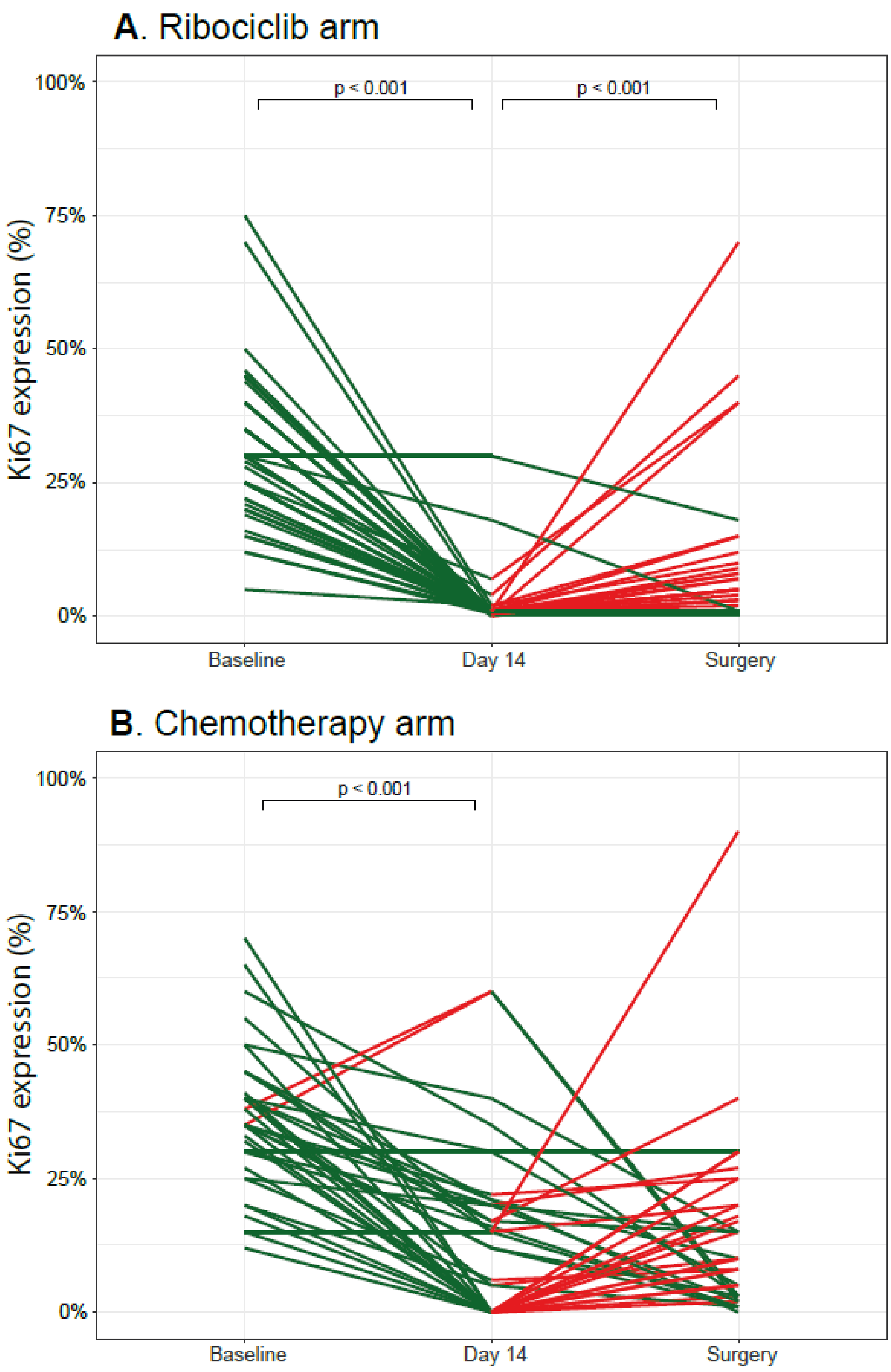

Supplementary Figure 3

Expression of Ki67 in surgical samples in patients achieving a response versus stable disease by magnetic resonance imaging (MRI) at 24 weeks of treatment. (A) of ribociclib and letrozole arm; (B) of chemotherapy. Expression of Ki67 in surgical samples in patients achieving a low PAM50-ROR vs intermediate vs high (C) after ribociclib and letrozole arm; (D) after chemotherapy. Correlation of Ki67 and PAM50-ROR in surgical samples after neoadjuvant (E) ribociclib and letrozole (F) after chemotherapy. Expression of Ki67 in surgical samples in patients achieving a PEPI score 0 vs 1-3 vs >4 (G) after ribociclib and letrozole arm; (H) after chemotherapy. Expression of Ki67 in surgical samples in patients achieving a residual cancer burden (RCB) 0 vs I vs II vs III (I) after ribociclib and letrozole arm; (J) after chemotherapy.

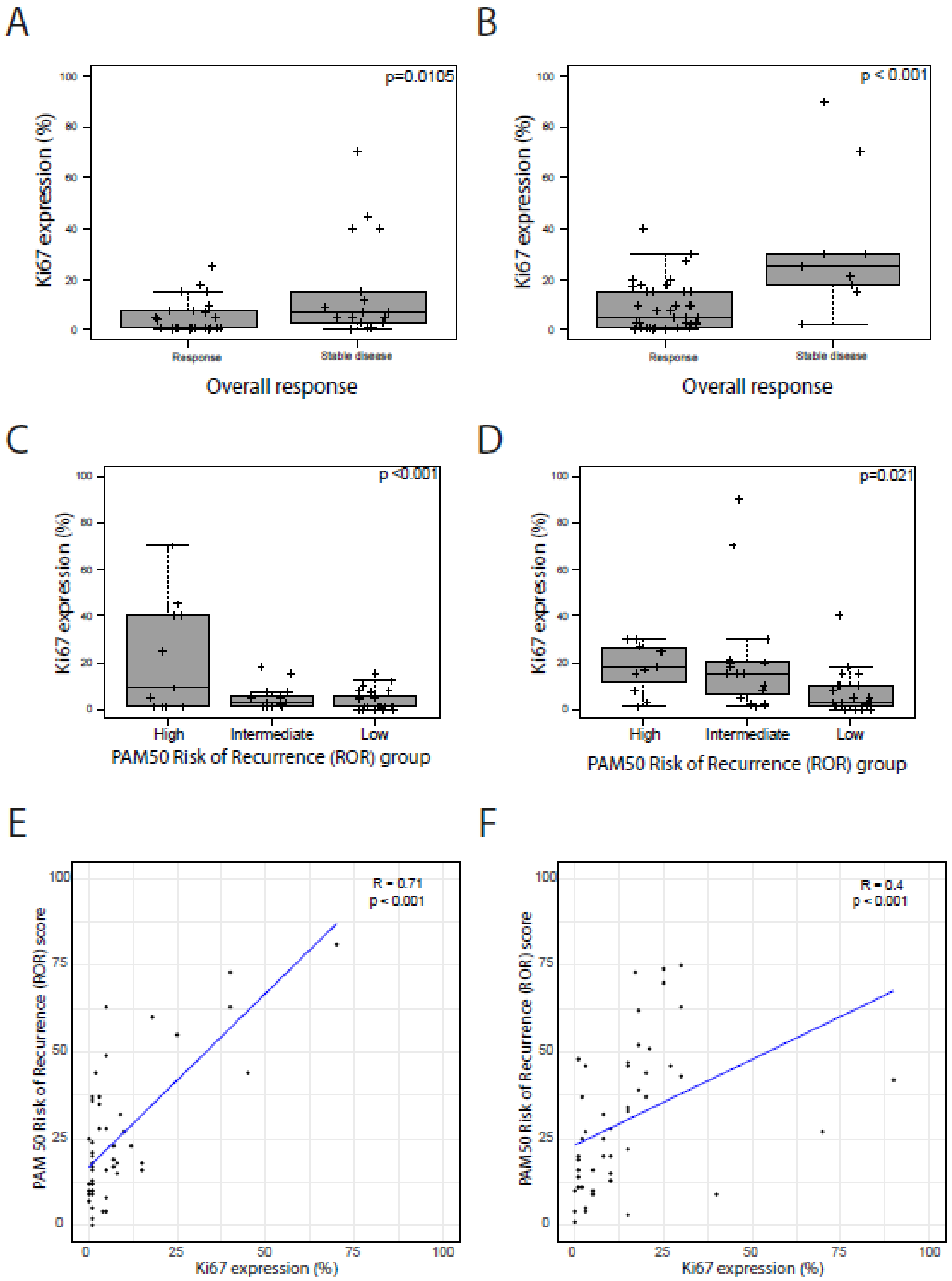

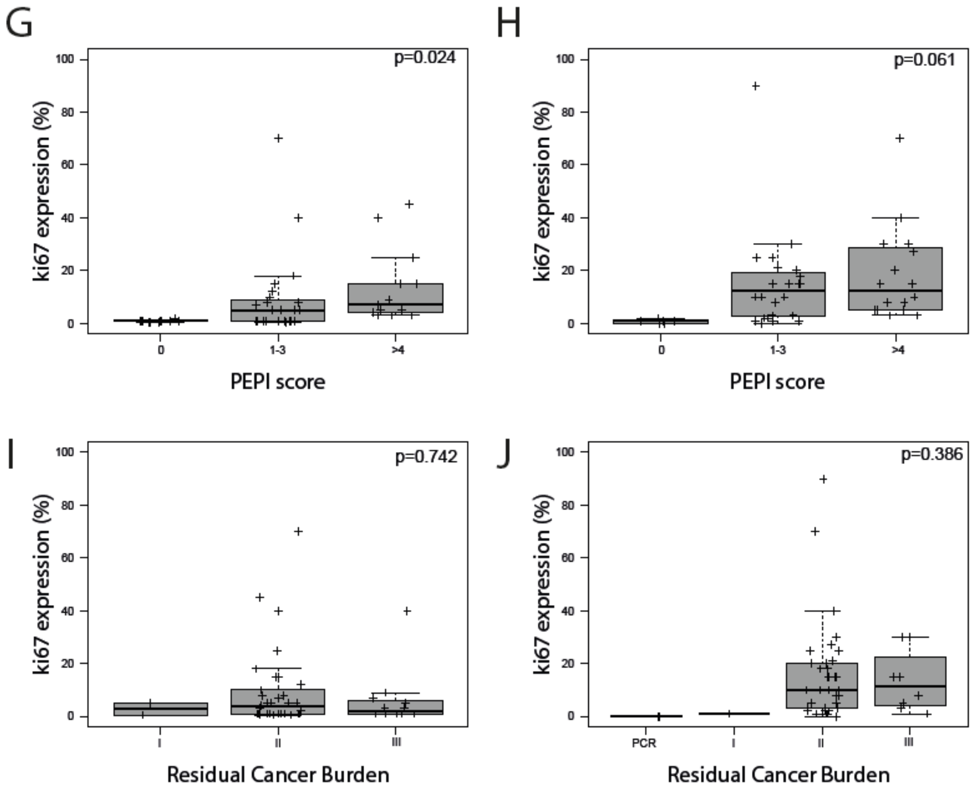
G
p=0.024
ki67 expression (%)
PEPI score
H
p=0.061
ki67 expression (%)
PEPI score
I
p=0.742
ki67 expression (%)
Residual Cancer Burden
J
p=0.386
ki67 expression (%)
Residual Cancer Burden

Supplementary Figure 4

Breast cancer molecular subtype shifts between baseline, day 14 and surgery paired samples in ribociclib and letrozole group (left) and Chemotherapy group (right).

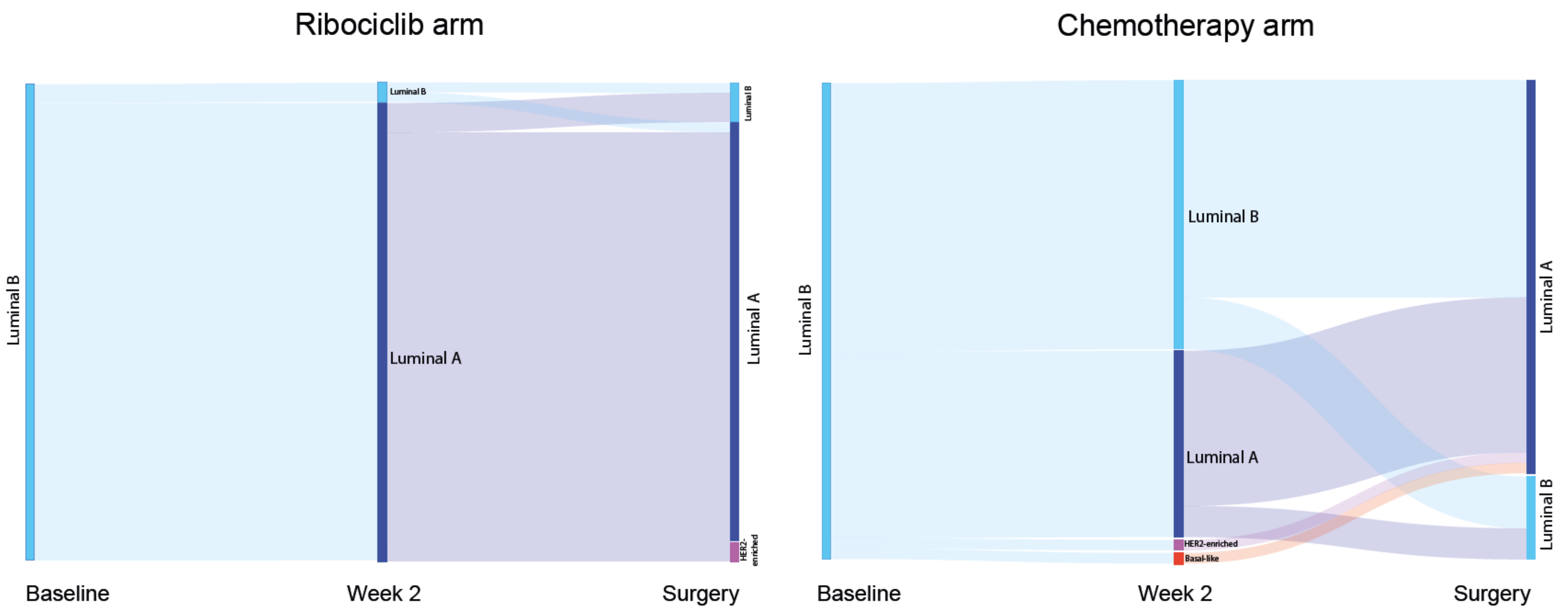

Supplementary Figure 5

A) Individual paired TILs levels at baseline and surgery after treatment with ribociclib and letrozole and multi-agent chemotherapy. Colored lines represent individual patient data. B) Individual TILs levels at baseline, day 14, and surgery. Colored lines represent individual patient data. C) TILs levels across the three timepoints (Baseline, day 14, surgery) in ribociclib and chemotherapy arm. p-value was obtained after performing ANOVA test. D) Changes in Ki67 levels across 3 time-points. (Left) Ribociclib and letrozole arm; (ERight) Chemotherapy. Green line reflects any decrease in TILs levels between 2 time-points and red line reflects any increase in TILs levels. p-value was obtained after performing Wilcoxon signed-rank test.

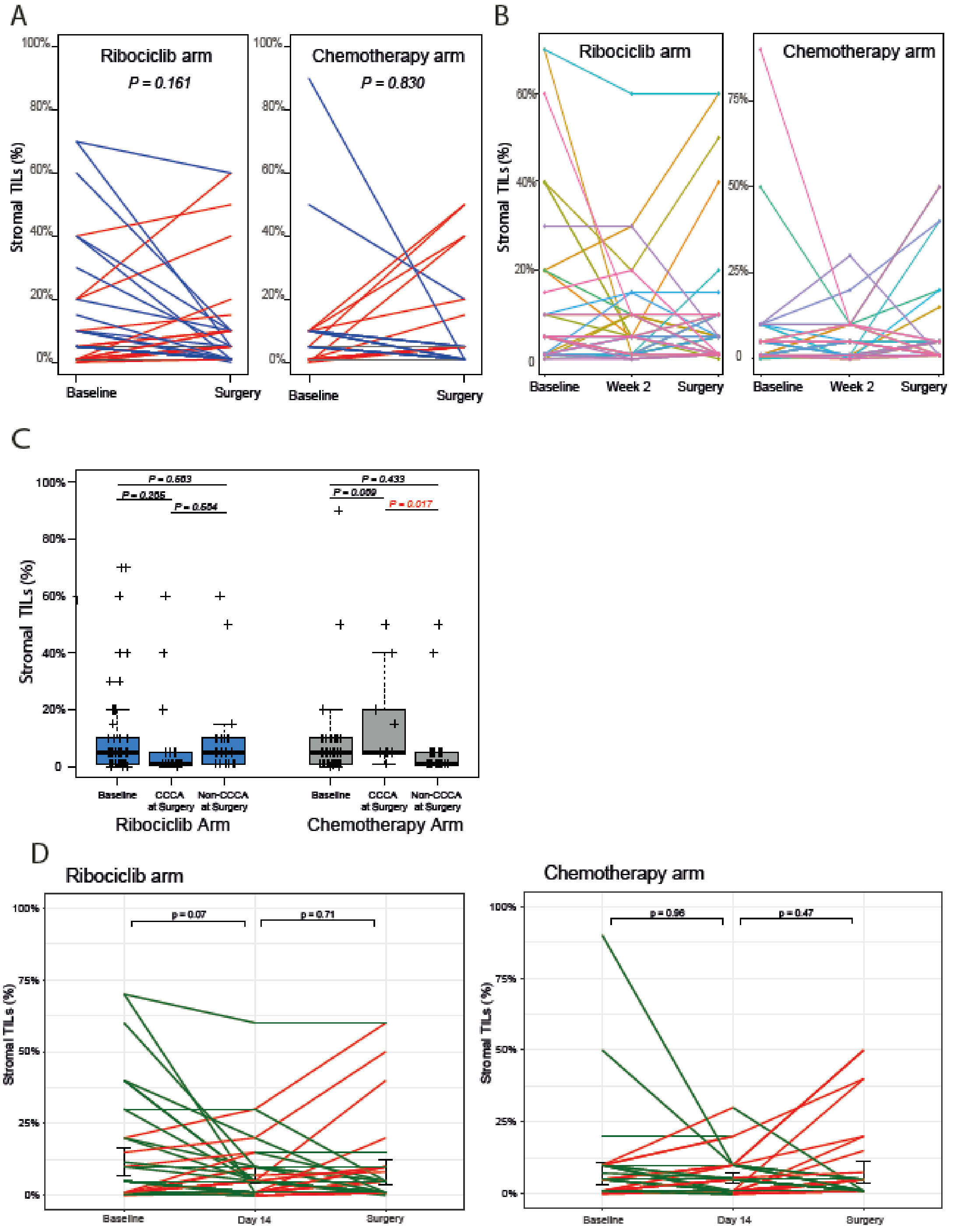

Supplementary Figure 6

Spearman correlation matrix for continuous TIL quantification and top 20 most correlated immune signatures, ordered by the correlation coefficients with TILs

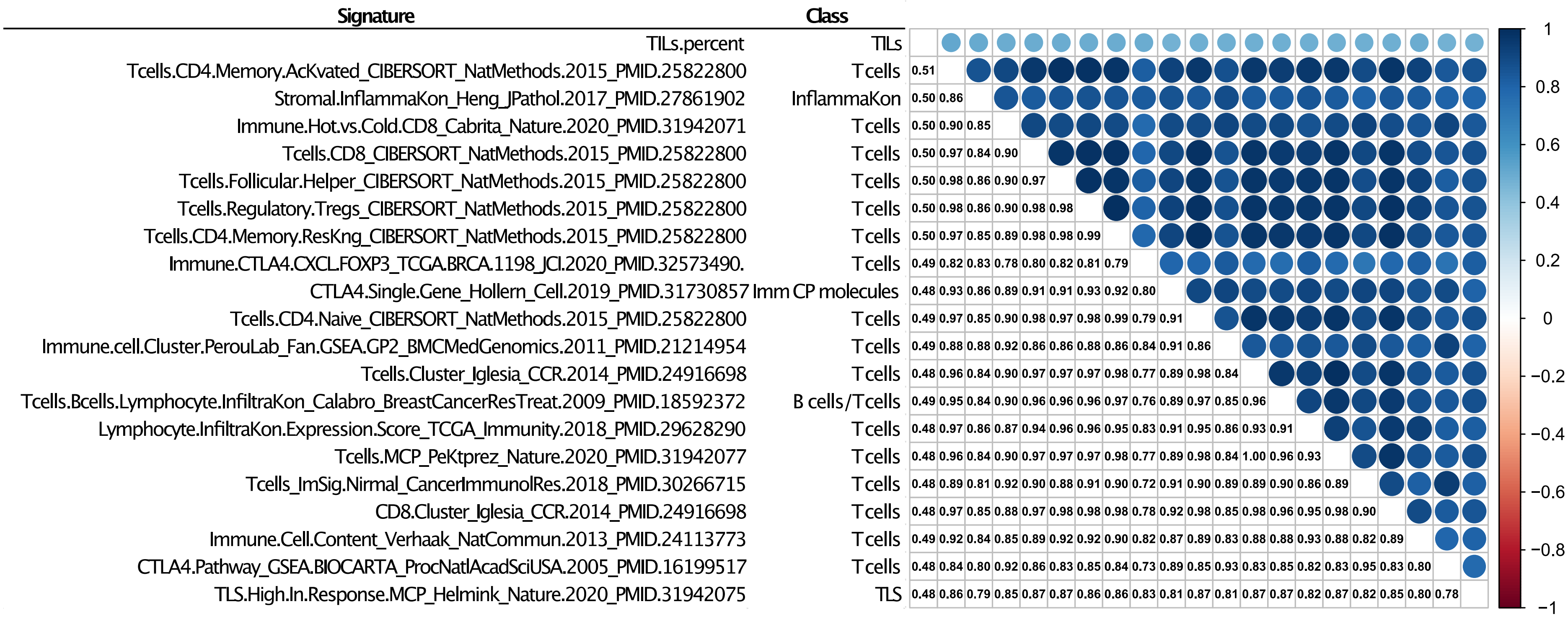

Supplemental Table 1. List and classification of immune gene expression signatures.

Signature_ID_paper	Immune_Class	Signature type	PMID
Bcells.Cluster_Iglesia_CCR.2014_PMID.24916698	B cells	Median signature	PMID.24916698
Bcells.IL10.Minus_Lin_JImmunol.2014_PMID.25080484	B cells	Median signature	PMID.25080484
Bcells.IL10.Plus_Lin_JImmunol.2014_PMID.25080484	B cells	Median signature	PMID.25080484
Bcells.Centroblast_Dybaer_JCO.2015_PMID.25800755	B cells	Median signature	PMID.25800755
Bcells.Centrocyte_Dybaer_JCO.2015_PMID.25800755	B cells	Median signature	PMID.25800755
Bcells.Memory_Dybaer_JCO.2015_PMID.25800755	B cells	Median signature	PMID.25800755
Bcells.Naive_Dybaer_JCO.2015_PMID.25800755	B cells	Median signature	PMID.25800755
Bcells.Memory_CIBERSORT_NatMethods.2015_PMID.25822800	B cells	Median signature	PMID.25822800
Bcells.Naive_CIBERSORT_NatMethods.2015_PMID.25822800	B cells	Median signature	PMID.25822800
Bcells_Garber_CellMolGastroenterolHepatol.2017_PMID.28508029	B cells	Median signature	PMID.28508029
Bcells.Extended_Garber_CellMolGastroenterolHepatol.2017_PMID.28508029	B cells	Median signature	PMID.28508029
Bcells_Bindea_Immunity.2013_PMID.24138885	B cells	Median signature	PMID.24138885
Bcells.ImmuneProfiles.Mouse.Human_Shay_PNAS.2013_PMID.23382184	B cells	Median signature	PMID.23382184
Bcells.Activated_Charoentong_CellRep.2017_PMID.28052254	B cells	Median signature	PMID.28052254
Bcells.Immature_Charoentong_CellRep.2017_PMID.28052254	B cells	Median signature	PMID.28052254
Bcells.Memory_Charoentong_CellRep.2017_PMID.28052254	B cells	Median signature	PMID.28052254
Bcells_ImSig.Nirmal_CancerImmunolRes.2018_PMID.30266715	B cells	Median signature	PMID.30266715
Bcells.Plasma.cells.52genes_Miller_GenomeBiol.2013_PMID.23618380	B cells	Median signature	PMID.23618380
Immune.CD19_TCGA.BRCA.1198_JCI.2020_PMID.32573490	B cells	Median signature	PMID.32573490

Signature_ID_paper	Immune_Class	Signature type	PMID
Immune1_TCGA.BRCA.1198_JCI.2020_PMID.32573490	B cells	Median signature	PMID.32573490
Bcells.Tcells.Cooperation_Hollern_Cell.2019_PMID.31730857	B cells/T cells	Median signature	PMID.31730857
Immune.14_Perez_JCO.2015_PMID.2560586	B cells/T cells	Median signature	PMID.25605861
Tcells.Bcells.Lymphocyte.Infiltration_Calabro_ BreastCancerResTreat.2009_PMID.18592372	B cells/T cells	Median signature	PMID.18592372
Immune.CD4.CD53.CD84.BTK_TCGA.BRCA.1198_JCI.2020_PMID.32573490	B cells/T cells	Median signature	PMID.32573490
CD103.Negative_Broz_CancerCell.2014_PMID.25446897	Dendritic cells	Median signature	PMID.25446897
Dendritic.cells.Activated_CIBERSORT_NatMethods.2015_PMID.25822800	Dendritic cells	Median signature	PMID.25822800
Dendritic.cells.Resting_CIBERSORT_NatMethods.2015_PMID.25822800	Dendritic cells	Median signature	PMID.25822800
Dendritic.cells.Activated_Bindea_Immunity.2013_PMID.24138885	Dendritic cells	Median signature	PMID.24138885
Dendritic.cells_Bindea_Immunity.2013_PMID.24138885	Dendritic cells	Median signature	PMID.24138885
Denditic.cells.ImmuneProfiles.Mouse.Human_Shay_PNAS.2013_PMID.23382184	Dendritic cells	Median signature	PMID.23382184
Dendritic.cells.Activated_Charoentong_CellRep.2017_PMID.28052254	Dendritic cells	Median signature	PMID.28052254
Dendritic.cells.Immature._Charoentong_CellRep.2017_PMID.28052254	Dendritic cells	Median signature	PMID.28052254
Dendritic.cells.Immature._Bindea_Immunity.2013_PMID.24138885	Dendritic cells	Median signature	PMID.24138885
Plasmacytoid.Dendritic.cell_Charoentong_CellRep.2017_PMID.28052254	Dendritic cells	Median signature	PMID.28052254
Eosinophils_CIBERSORT_NatMethods.2015_PMID.25822800	Eosinophils	Median signature	PMID.25822800
Eosinophils_Bindea_Immunity.2013_PMID.24138885	Eosinophils	Median signature	PMID.24138885
Eosinophils_Charoentong_CellRep.2017_PMID.28052254	Eosinophils	Median signature	PMID.28052254
Hematopoietic.Stem.cells.ImmuneProfiles.Mouse.Human_Shay_ PNAS.2013_PMID.23382184	Hematopoietic	Median signature	PMID.23382184
Immune.HLA.A.F_TCGA.BRCA.1198_JCI.2020_PMID.32573490	HLA	Median signature	PMID.32573490
Immune.HLA.D_TCGA.BRCA.1198_JCI.2020_PMID.32573490	HLA	Median signature	PMID.32573490
IFNa.Module10_Gatza_ProcNatlAcadSciUSA.2010_PMID.20335537	IFN	Median signature	PMID.20335537
IFNg.Module11_Gatza_ProcNatlAcadSciUSA.2010_PMID.20335537	IFN	Median signature	PMID.20335537

**Signature_ID_paper**	**Immune_Class**	**Signature type**	**PMID**
IFN.Cluster.GSEA.GP11_Fan_BMCMedGenomics.2011_PMID.21214954	**IFN**	Median signature	PMID.21214954
IFN_Rody_BreastCancerResearch.2008_PMID.19272155	**IFN**	Median signature	PMID.19272155
IFN.Cluster_Fan_BMCMedGenomics.2011_PMID.21214954	**IFN**	Median signature	PMID.21214954
IFN.3.ImmLandscape_Wolf_PlosOne.2014_PMID.24516633	**IFN**	Median signature	PMID.24516633
IFN.5.ImmLandscape_Wolf_PlosOne.2014_PMID.24516633	**IFN**	Median signature	PMID.24516633
IFN.Pathway_ImSig.Nirmal_CancerImmunolRes.2018_PMID.30266715	**IFN**	Median signature	PMID.30266715
IFN.Score.module3_TCGA_Immunity.2018_PMID.29628290	**IFN**	Median signature	PMID.29628290
Immune.IFN_TCGA.BRCA.1198_JCI.2020_PMID.32573490	**IFN**	Median signature	PMID.32573490
IgG_Rody_BreastCancerResearch.2008_PMID.19272155	**Ig**	Median signature	PMID.19272155
IGG.Cluster_Fan_BMCMedGenomics.2011_PMID.21214954	**Ig**	Median signature	PMID.21214954
Ig_TCGA.BRCA.1198_Cell.2015_PMID.26451490	**Ig**	Median signature	PMID.26451490
Immune.Suppression_Kardos_JCIInsight.2016_PMID.27699256	**Immune checkpoint molecules**	Median signature	PMID.27699256
CD274.Single.Gene_Hollern_Cell.2019_PMID.31730857	**Immune checkpoint molecules**	Single gene	PMID.31730857
CTLA4.Single.Gene_Hollern_Cell.2019_PMID.31730857	**Immune checkpoint molecules**	Single Gene	PMID.31730857
PDCD1.Single.Gene_Pare_AnnOncol.2019_PMID.30165419	**Immune checkpoint molecules**	Single Gene	PMID.30165419
Immunosuppression_Petitprez_Nature.2020_PMID.31942077	**Immune suppression**	Median signature	PMID.31942077
Stromal.Inflammation_Heng_JPathol.2017_PMID.27861902	**Inflammation**	Median signature	PMID.27861902
Wound.Healing_Chang_PlosBiol.2004_PMID.14737219	**Inflammation**	Median signature	PMID.14737219
TGFB.score_TCGA_Immunity.2018_PMID.29628290	**Inflammation**	Median signature	PMID.29628290
Immune.FOS.JUN.IL6_TCGA.BRCA.1198_JCI.2020_PMID.32573490	**Inflammation**	Median signature	PMID.32573490
Macrophages.M0_CIBERSORT_NatMethods.2015_PMID.25822800	**Macrophages**	Median signature	PMID.25822800
Macrophages.M1_CIBERSORT_NatMethods.2015_PMID.25822800	**Macrophages**	Median signature	PMID.25822800

Signature_ID_paper	Immune_Class	Signature type	PMID
Macrophages.M2_CIBERSORT_NatMethods.2015_PMID.25822800	Macrophages	Median signature	PMID.25822800
Macrophages_Bindea_Immunity.2013_PMID.24138885	Macrophages	Median signature	PMID.24138885
Macrophages.M2_Ghassabeh_Blood.2006_PMID.16556895	Macrophages	Median signature	PMID.16556895
Macrophages.Th1.Cluster_Iglesia_CCR.2014_PMID.24916698	Macrophages	Median signature	PMID.24916698
Macrophages.ImmuneProfiles.Mouse.Human_Shay_PNAS.2013_PMID.23382184	Macrophages	Median signature	PMID.23382184
Macrophages_Charoentong_CellRep.2017_PMID.28052254	Macrophages	Median signature	PMID.28052254
Macrophages.Monocytes.CSF1.Response_Beck_CCR.2009_PMID.19188147	Macrophages	Median signature	PMID. 19188147
Macrophages_ImSig.Nirmal_CancerImmunolRes.2018_PMID.30266715	Macrophages	Median signature	PMID.30266715
Immune.GIMAP.IL16_TCGA.BRCA.1198_JCI.2020_PMID.32573490	Macrophages	Median signature	PMID.32573490
Mast.cells.Activated_CIBERSORT_NatMethods.2015_PMID.25822800	Mastocytes	Median signature	PMID.25822800
Mast.cells.Resting_CIBERSORT_NatMethods.2015_PMID.25822800	Mastocytes	Median signature	PMID.25822800
Mast.cells_Bindea_Immunity.2013_PMID.24138885	Mastocytes	Median signature	PMID.24138885
Mast.cell_Charoentong_CellRep.2017_PMID.28052254	Mastocytes	Median signature	PMID.28052254
MDSC.Granulocytic_Youn_LeukocBiol.2012_PMID.21954284	MDSC	Median signature	PMID.21954284
MDSC.Neutrophil_Youn_LeukocBiol.2012_PMID.21954284	MDSC	Median signature	PMID.21954284
MDSC.Tumor.Macrophages_Schlecker_JImmunol.2012_PMID.23152559	MDSC	Median signature	PMID.23152559
MDSC.Tumor_Schlecker_JImmunol.2012_PMID.23152559	MDSC	Median signature	PMID.23152559
MDSC_Charoentong_CellRep.2017_PMID.28052254	MDSC	Median signature	PMID.28052254
MHC.11genes_Forero_CancerImmunolRes.2016_PMID.26980599	MHC	Median signature	PMID.26980599
MHC.24genes_Forero_CancerImmunolRes.2016_PMID.26980599	MHC	Median signature	PMID.26980599
MHC.I_Rody_BreastCancerResearch.2008_PMID.19272155	MHC	Median signature	PMID.19272155
MHC.II_Rody_BreastCancerResearch.2008_PMID.19272155	MHC	Median signature	PMID.19272155
MHC.I.CoreGenes_Lauss_NatCommun.2017_PMID29170503	MHC	Median signature	PMID.29170503
CD68.Cluster_Iglesia_CCR.2014_PMID.24916698	Monocytes	Median signature	PMID.24916698

**Signature_ID_paper**	**Immune_Class**	**Signature type**	**PMID**
Monocytes_CIBERSORT_NatMethods.2015_PMID.25822800	**Monocytes**	Median signature	PMID.25822800
Tcells.Effector.Memory_Bindea_Immunity.2013_PMID.24138885	**Monocytes**	Median signature	PMID.24138885
Monocytes.Dendritic.cell.Metagene_Miller_GenomeBiol.2013_PMID.23618380	**Monocytes**	Median signature	PMID.23618380
Monocytes_Charoentong_CellRep.2017_PMID.28052254	**Monocytes**	Median signature	PMID.28052254
Monocytes_ImSig.Nirmal_CancerImmunolRes.2018_PMID.30266715	**Monocytes**	Median signature	PMID.30266715
Monocytes.Dendritic.25genes_Miller_GenomeBiol.2013_PMID.23618380	**Monocytes**	Median signature	PMID.23618380
CSF1.Response_TCGA_Immunity.2018_PMID.29628290	**Monocytes**	Median signature	PMID.29628290
Immune.CD34.TIE1_TCGA.BRCA.1198_JCI.2020_PMID.32573490	**Monocytes**	Median signature	PMID.32573490
HCK_Rody_BreastCancerResearch.2008_PMID.19272155	**Monocytes**	Median signature	PMID.19272155
Influenza.11genes_Khatri_Immunity.2015_PMID.26682989	**Monocytes**	Median signature	PMID.26682989
Neutrophils.Activated.Blood_Janiszewska_NatCellBiol.2019_PMID.31263265	**Neutrophils**	Median signature	PMID.31263265
Neutrophils.Activated.Lung_Janiszewska_NatCellBiol.2019_PMID.31263265	**Neutrophils**	Median signature	PMID.31263265
Neutrophils_CIBERSORT_NatMethods.2015_PMID.25822800	**Neutrophils**	Median signature	PMID.25822800
Neutrophils_Bindea_Immunity.2013_PMID.24138885	**Neutrophils**	Median signature	PMID.24138885
Granulocytes.ImmuneProfiles.Mouse.Human_Shay_PNAS.2013_PMID.23382184	**Neutrophils**	Median signature	PMID.23382184
Neutrophils.MCP_Petitprez_Nature.2020_PMID.31942077	**Neutrophils**	Median signature	PMID.31942077
Neutrophils_Charoentong_CellRep.2017_PMID.28052254	**Neutrophils**	Median signature	PMID.28052254
Neutrophils_ImSig.Nirmal_CancerImmunolRes.2018_PMID.30266715	**Neutrophils**	Median signature	PMID.30266715
NK.Activated_CIBERSORT_NatMethods.2015_PMID.25822800	**NK cells**	Median signature	PMID.25822800
NK.Resting_CIBERSORT_NatMethods.2015_PMID.25822800	**NK cells**	Median signature	PMID.25822800
NK.CD56bright_Bindea_Immunity.2013_PMID.24138885	**NK cells**	Median signature	PMID.24138885
NK.CD56dim_Bindea_Immunity.2013_PMID.24138885	**NK cells**	Median signature	PMID.24138885
NK_Bindea_Immunity.2013_PMID.24138885	**NK cells**	Median signature	PMID.24138885
Tcells.Central.Memory_Bindea_Immunity.2013_PMID.24138885	**NK cells**	Median signature	PMID.24138885

**Signature_ID_paper**	**Immune_Class**	**Signature type**	**PMID**
NK.ImmuneProfiles.Mouse.Human_Shay_PNAS.2013_PMID.23382184	**NK cells**	Median signature	PMID.23382184
NK.CD56bright_Charoentong_CellRep.2017_PMID.28052254	**NK cells**	Median signature	PMID.28052254
NK.CD56dim_Charoentong_CellRep.2017_PMID.28052254	**NK cells**	Median signature	PMID.28052254
NK_Charoentong_CellRep.2017_PMID.28052254	**NK cells**	Median signature	PMID.28052254
NK.Tcell_Charoentong_CellRep.2017_PMID.28052254	**NK cells**	Median signature	PMID.28052254
NK_ImSig.Nirmal_CancerImmunolRes.2018_PMID.30266715	**NK cells**	Median signature	PMID.30266715
NK.MCP_Helmink_Nature.2020_PMID.31942077	**NK cells**	Median signature	PMID.31942077
Bcells.Plasmablast_Dybaer_JCO.2015_PMID.25800755	**Plasma cells**	Median signature	PMID.25800755
Plasma.cells_CIBERSORT_NatMethods.2015_PMID.25822800	**Plasma cells**	Median signature	PMID.25822800
Bcells.Plasma.cells.Metagene_Miller_GenomeBiol.2013_PMID.23618380	**Plasma cells**	Median signature	PMID.23618380
Plasma.cells_ImSig.Nirmal_CancerImmunolRes.2018_PMID.30266715	**Plasma cells**	Median signature	PMID.30266715
STAT1_Rody_BreastCancerResearch.2008_PMID.19272155	**STAT**	Median signature	PMID.19272155
Proliferation.Pathway_ImSig.Nirmal_CancerImmunolRes.2018_PMID.30266715	**T cells**	Median signature	PMID.30266715
Immune.CTLA4.CXCL.FOXP3_TCGA.BRCA.1198_JCI.2020_PMID.32573490.	**T cells**	Median signature	PMID.32573490
CD8.Cluster_Iglesia_CCR.2014_PMID.24916698	**T cells**	Median signature	PMID.24916698
Tcells.CD8.MCP_Petitprez_Nature.2020_PMID.31942077	**T cells**	Median signature	PMID.31942077
Tcells.CD4.Memory.Activated_CIBERSORT_NatMethods.2015_PMID.25822800	**T cells**	Median signature	PMID.25822800
Tcells.CD4.Memory.Resting_CIBERSORT_NatMethods.2015_PMID.25822800	**T cells**	Median signature	PMID.25822800
Tcells.CD4.Naive_CIBERSORT_NatMethods.2015_PMID.25822800	**T cells**	Median signature	PMID.25822800
Tcells.CD8_CIBERSORT_NatMethods.2015_PMID.25822800	**T cells**	Median signature	PMID.25822800
Tcells.Follicular.Helper_CIBERSORT_NatMethods.2015_PMID.25822800	**T cells**	Median signature	PMID.25822800
Tcells.Gamma.Delta_CIBERSORT_NatMethods.2015_PMID.25822800	**T cells**	Median signature	PMID.25822800
Tcells.Regulatory.Tregs_CIBERSORT_NatMethods.2015_PMID.25822800	**T cells**	Median signature	PMID.25822800
Cytolytic.Activity_Rooney_Cell.2015_PMID.25594174	**T cells**	Median signature	PMID.25594174

Signature_ID_paper	Immune_Class	Signature type	PMID
Cytotoxic.Lymphocytes.MCP_Petitprez_Nature.2020_PMID.31942077	T cells	Median signature	PMID.31942077
Immune.Active_Hollern_Cell.2019_PMID.31730857	T cells	Median signature	PMID.31730857
CTLA4.Pathway_GSEA.BIOCARTA_ProcNatlAcadSciUSA.2005_PMID.16199517	T cells	Median signature	PMID.16199517
Immune.cell.Cluster.PerouLab_Fan.GSEA.GP2_ BMCMedGenomics.2011_PMID.21214954	T cells	Median signature	PMID.16199517
Tcells.Bcell.KEGG.hematopoietic.cell.lineage_GSEA.GP2_ ProcNatlAcadSciUSA.2005_PMID.16199517	T cells	Median signature	PMID.16199517
PD1.Signaling.Reactome_GSEA_ProcNatlAcadSciUSA.2005_PMID.16199517	T cells	Median signature	PMID.16199517
Tcells.CD8_Bindea_Immunity.2013_PMID.24138885	T cells	Median signature	PMID.24138885
Cytotoxic.cells_Bindea_Immunity.2013_PMID.24138885	T cells	Median signature	PMID.24138885
Tcells_Bindea_Immunity.2013_PMID.24138885	T cells	Median signature	PMID.24138885
Tcells.Thelper_Bindea_Immunity.2013_PMID.24138885	T cells	Median signature	PMID.24138885
Tcells.Follicular.Helper_Bindea_Immunity.2013_PMID.24138885	T cells	Median signature	PMID.24138885
Tcells.Gamma.Delta_Bindea_Immunity.2013_PMID.24138885	T cells	Median signature	PMID.24138885
Tcells.Th1.cells_Bindea_Immunity.2013_PMID.24138885	T cells	Median signature	PMID.24138885
Tcells.Th17.cells_Bindea_Immunity.2013_PMID.24138885	T cells	Median signature	PMID.24138885
Tcells.Th2.cells_Bindea_Immunity.2013_PMID.24138885	T cells	Median signature	PMID.24138885
Immune.Hot.vs.Cold.CD8_Cabrita_Nature.2020_PMID.31942071	T cells	Median signature	PMID.31942071
Immune.87_Perez_JCO.2015_PMID.2560586	T cells	Median signature	PMID.2560586
LCK_Rody_BreastCancerResearch.2008_PMID.19272155	T cells	Median signature	PMID.19272155
MCD3.CD8_Fan_BMCMedGenomics.2011_PMID.21214954	T cells	Median signature	PMID.21214954
MERCK.Immune.Signature_Cristescu_Science.2018_PMID.30309915	T cells	Median signature	PMID.30309915
Tcells.NK.Metagene_Miller_GenomeBiol.2013_PMID.23618380	T cells	Median signature	PMID.23618380
Tcells.ImmuneProfiles.Mouse.Human_Shay_PNAS.2013_PMID.23382184_	T cells	Median signature	PMID.23382184
Tcells.CD4.Activated_Charoentong_CellRep.2017_PMID.28052254	T cells	Median signature	PMID.28052254

Signature_ID_paper	Immune_Class	Signature type	PMID
Tcells.CD8.Activated_Charoentong_CellRep.2017_PMID.28052254	**T cells**	Median signature	PMID.28052254
Tcells.CD4.Central.Memory_Charoentong_CellRep.2017_PMID.28052254	**T cells**	Median signature	PMID.28052254
Tcells.CD8.Central.Memory_Charoentong_CellRep.2017_PMID.28052254	**T cells**	Median signature	PMID.28052254
Tcells.CD4.Effector.Memory_Charoentong_CellRep.2017_PMID.28052254	**T cells**	Median signature	PMID.28052254
Tcells.CD8.Effector.Memory_Charoentong_CellRep.2017_PMID.28052254	**T cells**	Median signature	PMID.28052254
Tcells.Gamma.Delta_Charoentong_CellRep.2017_PMID.28052254	**T cells**	Median signature	PMID.28052254
Tcells.Regulatory_Charoentong_CellRep.2017_PMID.28052254	**T cells**	Median signature	PMID.28052254
Tcells.Follicular.Helper_Charoentong_CellRep.2017_PMID.28052254	**T cells**	Median signature	PMID.28052254
Tcells.Th1.cells_Charoentong_CellRep.2017_PMID.28052254	**T cells**	Median signature	PMID.28052254
Tcells.Th17.cells_Charoentong_CellRep.2017_PMID.28052254	**T cells**	Median signature	PMID.28052254
Tcells.Th2_Charoentong_CellRep.2017_PMID.28052254	**T cells**	Median signature	PMID.28052254
Tcells_ImSig.Nirmal_CancerImmunolRes.2018_PMID.30266715	**T cells**	Median signature	PMID.30266715
Tcells.NK.51genes_Miller_GenomeBiol.2013_PMID.23618380	**T cells**	Median signature	PMID.23618380
Tcells.CD8.Effector.vs.Naive.2_Pauken_Science.2016_PMID.27789795	**T cells**	Median signature	PMID.27789795
Tcells.CD8.Exhausted.vs.AntiPDL1.2_Pauken_Science.2016_PMID.27789795	**T cells**	Median signature	PMID.27789795
Tcells.CD8.Exhausted.vs.Naive.2_Pauken_Science.2016_PMID.27789795	**T cells**	Median signature	PMID.27789795
Tcells.CD8.Memory.vs.Naive.1_Pauken_Science.2016_PMID.27789795	**T cells**	Median signature	PMID.27789795
Tcells.Activation_Petitprez_Nature.2020_PMID.31942077	**T cells**	Median signature	PMID.31942077
Tcells.Cluster_Iglesia_CCR.2014_PMID.24916698	**T cells**	Median signature	PMID.24916698
Tcells.Survival.2gene_Petitprez_Nature.2020_PMID.31942077	**T cells**	Median signature	PMID.31942077
Tcells.Regulatory.cell.2gene_Petitprez_Nature.2020_PMID.31942077	**T cells**	Median signature	PMID.31942077
Tcells.Resident.Memory.Single.cell_Savas_NatMed.2018_PMID.29942092	**T cells**	Median signature	PMID.29942092
Tcells.MCP_Petitprez_Nature.2020_PMID.31942077	**T cells**	Median signature	PMID.31942077
Tcells.CD8.Exhausted.Anti.PDL1.vs.Control.Metagene.1_ Pauken_Science.2016_PMID.27789795	**T cells**	Median signature	PMID.27789795

Signature_ID_paper	Immune_Class	Signature type	PMID
Tcells.CD8.Exhausted.at.day.8.post.Imm.vs.Naive.Metagene.1_ Pauken_Science.2016_PMID.27789795	T cells	Median signature	PMID.27789795
Tcells.CD8.Exhausted.vs.Naive.Metagene.1_Pauken_Science.2016_PMID.27789795	T cells	Median signature	PMID.27789795
Tcells.CD8.Exhausted.vs.Naive.Metagene.3_Pauken_Science.2016_PMID.27789795	T cells	Median signature	PMID.27789795
Tcells.CD8.Memory.vs.Naive.Metagene.1_Pauken_Science.2016_PMID.27789795	T cells	Median signature	PMID.27789795
Tcells.CD8.Memory.vs.Naive.Metagene.2_Pauken_Science.2016_PMID.27789795	T cells	Median signature	PMID.27789795
Tcells.CD8.Memory.vs.Naive.Metagene.3_Pauken_Science.2016_PMID.27789795	T cells	Median signature	PMID.27789795
Serum.Response.Up_TCGA_Immunity.2018_PMID.29628290	T cells	Median signature	PMID.29628290
Lymphocyte.Infiltration.Expression.Score_TCGA_Immunity.2018_PMID.29628290	T cells	Median signature	PMID.29628290
Tcells.Follicular.Helper_TCGA_Immunity.2018_PMID.29628290	T cells	Median signature	PMID.29628290
Tcells.Gamma.Delta_TCGA_Immunity.2018_PMID.29628290	T cells	Median signature	PMID.29628290
Immune.CD8.GZMK_TCGA.BRCA.1198_JCI.2020_PMID.32573490	T cells	Median signature	PMID.32573490
Immune.Cell.Content_Verhaak_NatCommun.2013_PMID.24113773	T cells	Median signature	PMID.24113773
TLS.Hallmark_Cabrita_Nature.2020_PMID.31942071	TLS	Median signature	PMID.31942071
TLS.Known.Markers_Cabrita_Nature.2020_PMID.31942071	TLS	Median signature	PMID.31942071
TLS.12genes.Chemokine_Zhu_FrontImmunol.2017_PMID.28713385	TLS	Median signature	PMID.28713385
TLS.High.In.No.Response_Helmink_Nature.2020_PMID.31942075	TLS	Median signature	PMID.31942075
TLS.High.In.Response.MCP_Helmink_Nature.2020_PMID.31942075	TLS	Median signature	PMID.31942075
TLS.9genes_Cabrita_Nature.2020_PMID.31942071	TLS	Median signature	PMID.31942071
TLS.Tumors.w.TLS.and.CD8.vs.CD8.alone_Cabrita_Nature.2020_PMID.31942071	TLS	Median signature	PMID.31942071

Supplemental Table 2. List of non-immune gene expression signatures.

**Signature_ID_paper**	**Signature type**	**PMID**
HouseKeeping_Genome.Biol.2004_PMID.15287981	Median signature	PMID.15287981
GATA3.induced.genes_Oncogene.2004_PMID.15361840	Median signature	PMID.15361840
MCF7.E2.induced.genes_JCO.2006_PMID.16505416	Median signature	PMID.16505416
GATA3.induced.genes_JCO.2006_PMID.16505416	Median signature	PMID.16505416
MCF7.E2.repressed.genes_JCO.2006_PMID.16505416	Median signature	PMID.16505416
CIN70_Nat.Genet.2006_PMID.16921376	Median signature	PMID.16921376
RB_LOSS_J.Clin.Invest.2007_PMID.17160137	Median signature	PMID.17160137
Shipitsin_CD44_A_Cancer.Cell.2007_PMID.17349583	Median signature	PMID.17349583
Shipitsin_CD44_B_Cancer.Cell.2007_PMID.17349583	Median signature	PMID.17349583
KRAS_amplicon_Genome.Biology.2007_PMID.17493263	Median signature	PMID.17493263
Claudin_High_Genome.Biol.2007_PMID.17493263	Median signature	PMID.17493263
MM_p53null_1pFDR_UP_Genome.Biology.2007_PMID.17493263	Median signature	PMID.17493263
Claudin_Low_Genome.Biol.2007_PMID.17493263	Median signature	PMID.17493263
MM_DMBAwnt_1pFDR_UP_Genome.Biology.2007_PMID.17493263	Median signature	PMID.17493263
GSEA_GP5_MYC_targets_TERT.r=0.922_PerouLab_MM_Myc_1pFDR_UP_ Genome_Biology_2007_PMID.17493263	Median signature	PMID.17493263
MM_NeuPyMT_1pFDR_UP_Genome.Biology.2007_PMID.17493263	Median signature	PMID.17493263
MM_C3Tag_1pFDR_UP_Genome.Biology.2007_PMID.17493263	Median signature	PMID.17493263
MM_WapINT3_1pFDR_UP_Genome.Biology.2007_PMID.17493263	Median signature	PMID.17493263
MM_BRCAwnt_1pFDR_UP_Genome.Biology.2007_PMID.17493263	Median signature	PMID.17493263
MM_WAPTag_1pFDR_UP_Genome.Biology.2007_PMID.17493263	Median signature	PMID.17493263

**Signature_ID_paper**	**Signature type**	**PMID**
MM_Potluck_1pFDR_UP_Genome.Biology.2007_PMID.17493263	Median signature	PMID.17493263
MM_Normal_1pFDR_UP_Genome.Biology.2007_PMID.17493263	Median signature	PMID.17493263
HER1_Cluster2_BMC.Genomics.2007_PMID.17663798	Median signature	PMID.17663798
HER1_Cluster1_BMC.Genomics.2007_PMID.17663798	Median signature	PMID.17663798
HER1_Cluster3_BMC.Genomics.2007_PMID.17663798	Median signature	PMID.17663798
Fibromatosis_Lab.Invest.2008_PMID.18414401	Median signature	PMID.18414401
Murat_G24_J.Clin.Oncol.2008_PMID.18565887	Median signature	PMID.18565887
Murat_G18_J.Clin.Oncol.2008_PMID.18565887	Median signature	PMID.18565887
Murat_G07_J.Clin.Oncol.2008_PMID.18565887	Median signature	PMID.18565887
Wirapati_Proliferation_Breast.Cancer.Res.2008_PMID.18662380	Median signature	PMID.18662380
RB_LOH_Breast.Cancer.Res.2008_PMID.18782450	Median signature	PMID.18782450
bMYB_Signature_Oncogene.2009_PMID.19043454	Median signature	PMID.19043454
Glycolysis_Signature_BMC.Med.2009_PMID.19291283	Median signature	PMID.19291283
VEGF_13genes_BMC.Med.2009_PMID.19291283	Median signature	PMID.19291283
Claudin_Low_29_Cancer.Res.2009_PMID.19435916	Median signature	PMID.19435916
METAPLASTIC_Up_CanRes.2009_PMID.19435916	Median signature	PMID.19435916
Lung.WNT.DiMeo_Cancer.Res.2009_PMID.19549913	Median signature	PMID.19549913
Luminal_Progenitor_Down_Nat.Med.2009_PMID.19648928	Median signature	PMID.19648928
Mature_LuminaUp_Nat.Med.2009_PMID.19648928	Median signature	PMID.19648928
Mature_Luminal_Down_Nat.Med.2009_PMID.19648928	Median signature	PMID.19648928
Stromal_Up_Nat.Med.2009_PMID.19648928	Median signature	PMID.19648928
MASC_Down_Nat.Med.2009_PMID.19648928	Median signature	PMID.19648928
Stromal_Down_Nat.Med.2009_PMID.19648928	Median signature	PMID.19648928
Luminal_Progenitor_Up_Nat.Med.2009_PMID.19648928	Median signature	PMID.19648928
MASC_Up_Nat.Med.2009_PMID.19648928	Median signature	PMID.19648928

**Signature_ID_paper**	**Signature type**	**PMID**
MS_CD44_UP_PNAS.2009_PMID.19666588	Median signature	PMID.19666588
MS_CD44_DOWN_PNAS.2009_PMID.19666588	Median signature	PMID.19666588
Troester_Wound_Signature_Clin.Cancer.Res.2009_PMID.19887484	Median signature	PMID.19887484
Duke_Module17_pr_Mike_PMID.20335537	Median signature	PMID.20335537
Duke_Module02_akt_Mike_PMID.20335537	Median signature	PMID.20335537
Duke_Module05_egfr_Mike_PMID.20335537	Median signature	PMID.20335537
Duke_Module09_hypoxia_Mike_PMID.20335537	Median signature	PMID.20335537
Duke_Module22_tnfa_Mike_PMID.20335537	Median signature	PMID.20335537
Duke_Module12_lacticacidosis_Mike_PMID.20335537	Median signature	PMID.20335537
Duke_Module07_glucosedepletion_Mike_PMID.20335537	Median signature	PMID.20335537
Duke_Module04_e2f1_Mike_PMID.20335537	Median signature	PMID.20335537
Duke_Module16_pi3k_Mike_PMID.20335537	Median signature	PMID.20335537
Duke_Module14_p53_Mike_PMID.20335537	Median signature	PMID.20335537
Duke_Module06_er_Mike_PMID.20335537	Median signature	PMID.20335537
Duke_Module15_p63_Mike_PMID.20335537	Median signature	PMID.20335537
Duke_Module13_myc_Mike_PMID.20335537	Median signature	PMID.20335537
Duke_Module19_src_Mike_PMID.20335537	Median signature	PMID.20335537
Duke_Module03_betacatenin_Mike_PMID.20335537	Median signature	PMID.20335537
Duke_Module01_acidosis_Mike_PMID.20335537	Median signature	PMID.20335537
Duke_Module21_tgfb_Mike_PMID.20335537	Median signature	PMID.20335537
Duke_Module08_her2_Mike_PMID.20335537	Median signature	PMID.20335537
Lim.et.al.2010.Conserved.Mature_BCR.2010_PMID.20346151	Median signature	PMID.20346151
Lim.et.al.2010.Conserved.Luminal.Progenitor_BCR.2010_PMID.20346151	Median signature	PMID.20346151
Lim.et.al.2010.Conserved.aMaSC_BCR.2010_PMID.20346151	Median signature	PMID.20346151
EMT_DOWN_Weingberg_PNAS.2010_PMID.20713713	Median signature	PMID.20713713

**Signature_ID_paper**	**Signature type**	**PMID**
EMT_UP_Weinberg_PNAS.2010_PMID.20713713	Median signature	PMID.20713713
Taube_EMT_down_PNAS.2010_PMID.20713713	Median signature	PMID.20713713
Taube_EMT_up_PNAS.2010_PMID.20713713	Median signature	PMID.20713713
MDACC.FNA.1_J.Clin.Oncol.2010_PMID.20805453	Median signature	PMID.20805453
MDACC.FNA.2_J.Clin.Oncol.2010_PMID.20805453	Median signature	PMID.20805453
HRneg.Tneg_14.Genes_BCR.2010_PMID.20946665	Median signature	PMID.20946665
C_MYB_Signature_PLoS.One.2010_PMID.20949095	Median signature	PMID.20949095
MM_Red3_BMC.Med.Genomics.2011_PMID.21214954	Median signature	PMID.21214954
MM_Red5_BMC.Med.Genomics.2011_PMID.21214954	Median signature	PMID.21214954
MM_Red7_BMC.Med.Genomics.2011_PMID.21214954	Median signature	PMID.21214954
MM_Green13_BMC.Med.Genomics.2011_PMID.21214954	Median signature	PMID.21214954
MM_Red2_BMC.Med.Genomics.2011_PMID.21214954	Median signature	PMID.21214954
MM_Red9_BMC.Med.Genomics.2011_PMID.21214954	Median signature	PMID.21214954
MM_Green11_BMC.Med.Genomics.2011_PMID.21214954	Median signature	PMID.21214954
MM_Red19_BMC.Med.Genomics.2011_PMID.21214954	Median signature	PMID.21214954
MM_Green2_BMC.Med.Genomics.2011_PMID.21214954	Median signature	PMID.21214954
MM_Red16_BMC.Med.Genomics.2011_PMID.21214954	Median signature	PMID.21214954
MM_Green8_BMC.Med.Genomics.2011_PMID.21214954	Median signature	PMID.21214954
MM_Red8_BMC.Med.Genomics.2011_PMID.21214954	Median signature	PMID.21214954
4p16_Amplicon_BMC.Med.Genomics.2011_PMID.21214954	Median signature	PMID.21214954
MM_Green18_BMC.Med.Genomics.2011_PMID.21214954	Median signature	PMID.21214954
MM_Green16_BMC.Med.Genomics.2011_PMID.21214954	Median signature	PMID.21214954
MM_Red22_BMC.Med.Genomics.2011_PMID.21214954	Median signature	PMID.21214954
MM_Green9_BMC.Med.Genomics.2011_PMID.21214954	Median signature	PMID.21214954
MM_Red12_BMC.Med.Genomics.2011_PMID.21214954	Median signature	PMID.21214954

Signature_ID_paper	Signature type	PMID
MM_Green15_BMC.Med.Genomics.2011_PMID.21214954	Median signature	PMID.21214954
MM_Red20_BMC.Med.Genomics.2011_PMID.21214954	Median signature	PMID.21214954
MM_Green6_BMC.Med.Genomics.2011_PMID.21214954	Median signature	PMID.21214954
MM_Green7_BMC.Med.Genomics.2011_PMID.21214954	Median signature	PMID.21214954
MM_Green3_BMC.Med.Genomics.2011_PMID.21214954	Median signature	PMID.21214954
MM_Red25_BMC.Med.Genomics.2011_PMID.21214954	Median signature	PMID.21214954
MM_Green20_BMC.Med.Genomics.2011_PMID.21214954	Median signature	PMID.21214954
MVEGFC_BMC.Med.Genomics.2011_PMID.21214954	Median signature	PMID.21214954
MM_Green24_BMC.Med.Genomics.2011_PMID.21214954	Median signature	PMID.21214954
MKRAS_amplicon_BMC.Med.Genomics.2011_PMID.21214954	Median signature	PMID.21214954
MITO1_BMC.Med.Genomics.2011_PMID.21214954	Median signature	PMID.21214954
MM_Red21_BMC.Med.Genomics.2011_PMID.21214954	Median signature	PMID.21214954
MM_Green21_BMC.Med.Genomics.2011_PMID.21214954	Median signature	PMID.21214954
HS_Red5_BMC.Med.Genomics.2011_PMID.21214954	Median signature	PMID.21214954
HS_Green10_BMC.Med.Genomics.2011_PMID.21214954	Median signature	PMID.21214954
HS_Green4_BMC.Med.Genomics.2011_PMID.21214954	Median signature	PMID.21214954
HS_Red10_BMC.Med.Genomics.2011_PMID.21214954	Median signature	PMID.21214954
HS_Red3_BMC.Med.Genomics.2011_PMID.21214954	Median signature	PMID.21214954
GSEA_GP4_MES_ECM.r=0.954_PerouLab_HS_Red7_ BMC_Med_Genomics_2011_PMID.21214954	Median signature	PMID.21214954
MM_Green14_BMC.Med.Genomics.2011_PMID.21214954	Median signature	PMID.21214954
MM_Red11_BMC.Med.Genomics.2011_PMID.21214954	Median signature	PMID.21214954
MM_Red4_BMC.Med.Genomics.2011_PMID.21214954	Median signature	PMID.21214954
MM_Red18_BMC.Med.Genomics.2011_PMID.21214954	Median signature	PMID.21214954
MM_Red24_BMC.Med.Genomics.2011_PMID.21214954	Median signature	PMID.21214954

**Signature_ID_paper**	**Signature type**	**PMID**
AMPH_EPIREGULIN_Cluster_BMC.Med.Genomics.2011_PMID.21214954	Median signature	PMID.21214954
Mmyosin_BMC.Med.Genomics.2011_PMID.21214954	Median signature	PMID.21214954
Unknown_13_BMC.Med.Genomics.2011_PMID.21214954	Median signature	PMID.21214954
17PP13_Amplicon_BMC.Med.Genomics.2011_PMID.21214954	Median signature	PMID.21214954
MHistone_BMC.Med.Genomics.2011_PMID.21214954	Median signature	PMID.21214954
HISTONE_BMC.Med.Genomics.2011_PMID.21214954	Median signature	PMID.21214954
HS_Green23_BMC.Med.Genomics.2011_PMID.21214954	Median signature	PMID.21214954
Unknown_5_BMC.Med.Genomics.2011_PMID.21214954	Median signature	PMID.21214954
FOS_JUN_Cluster_BMC.Med.Genomics.2011_PMID.21214954	Median signature	PMID.21214954
Unknown_3_BMC.Med.Genomics.2011_PMID.21214954	Median signature	PMID.21214954
CD34_CD36_Cluster_BMC.Med.Genomics.2011_PMID.21214954	Median signature	PMID.21214954
Unknown_15_BMC.Med.Genomics.2011_PMID.21214954	Median signature	PMID.21214954
MAGE_Cluster_BMC.Med.Genomics.2011_PMID.21214954	Median signature	PMID.21214954
Secretoglobin_BMC.Med.Genomics.2011_PMID.21214954	Median signature	PMID.21214954
MClaudin_Cluster_BMC.Med.Genomics.2011_PMID.21214954	Median signature	PMID.21214954
GSEA_GP9_Cell_cell_adhesion.r=960_PerouLab_MClaudin_Cluster_ BMC_Med_Genomics_2011_PMID.21214954	Median signature	PMID.21214954
BASAL_Cluster_BMC.Med.Genomics.2011_PMID.21214954	Median signature	PMID.21214954
MRibosomal_BMC.Med.Genomics.2011_PMID.21214954	Median signature	PMID.21214954
Ribosomal_Cluster_BMC.Med.Genomics.2011_PMID.21214954	Median signature	PMID.21214954
Chromogramin_BMC.Med.Genomics.2011_PMID.21214954	Median signature	PMID.21214954
Unknown_1_BMC.Med.Genomics.2011_PMID.21214954	Median signature	PMID.21214954
16.13_Amplicon_BMC.Med.Genomics.2011_PMID.21214954	Median signature	PMID.21214954
8p_Amplicon_BMC.Med.Genomics.2011_PMID.21214954	Median signature	PMID.21214954
19p13_Amplicon_BMC.Med.Genomics.2011_PMID.21214954	Median signature	PMID.21214954

**Signature_ID_paper**	**Signature type**	**PMID**
MM_Green25_BMC.Med.Genomics.2011_PMID.21214954	Median signature	PMID.21214954
Unknown_4_BMC.Med.Genomics.2011_PMID.21214954	Median signature	PMID.21214954
HER2_Amplicon_BMC.Med.Genomics.2011_PMID.21214954	Median signature	PMID.21214954
MPYMT_NEU_Cluster_BMC.Med.Genomics.2011_PMID.21214954	Median signature	PMID.21214954
Fibrinogen_Cluster_BMC.Med.Genomics.2011_PMID.21214954	Median signature	PMID.21214954
MM_Green23_BMC.Med.Genomics.2011_PMID.21214954	Median signature	PMID.21214954
MM_Red10_BMC.Med.Genomics.2011_PMID.21214954	Median signature	PMID.21214954
MM_Red6_BMC.Med.Genomics.2011_PMID.21214954	Median signature	PMID.21214954
MM_Green1_BMC.Med.Genomics.2011_PMID.21214954	Median signature	PMID.21214954
MM_Green17_BMC.Med.Genomics.2011_PMID.21214954	Median signature	PMID.21214954
Unknown_6_BMC.Med.Genomics.2011_PMID.21214954	Median signature	PMID.21214954
HS_Red13_BMC.Med.Genomics.2011_PMID.21214954	Median signature	PMID.21214954
MM_Green4_BMC.Med.Genomics.2011_PMID.21214954	Median signature	PMID.21214954
MM_Green5_BMC.Med.Genomics.2011_PMID.21214954	Median signature	PMID.21214954
HS_Green15_BMC.Med.Genomics.2011_PMID.21214954	Median signature	PMID.21214954
HS_Green3_BMC.Med.Genomics.2011_PMID.21214954	Median signature	PMID.21214954
HS_Red9_BMC.Med.Genomics.2011_PMID.21214954	Median signature	PMID.21214954
HS_Green1_BMC.Med.Genomics.2011_PMID.21214954	Median signature	PMID.21214954
HS_Green7_BMC.Med.Genomics.2011_PMID.21214954	Median signature	PMID.21214954
HS_Red2_BMC.Med.Genomics.2011_PMID.21214954	Median signature	PMID.21214954
HS_Green5_BMC.Med.Genomics.2011_PMID.21214954	Median signature	PMID.21214954
HS_Red23_BMC.Med.Genomics.2011_PMID.21214954	Median signature	PMID.21214954
HS_Red6_BMC.Med.Genomics.2011_PMID.21214954	Median signature	PMID.21214954
HS_Green11_BMC.Med.Genomics.2011_PMID.21214954	Median signature	PMID.21214954
HS_Red17_BMC.Med.Genomics.2011_PMID.21214954	Median signature	PMID.21214954

Signature_ID_paper	Signature type	PMID
HS_Green16_BMC.Med.Genomics.2011_PMID.21214954	Median signature	PMID.21214954
HS_Red11_BMC.Med.Genomics.2011_PMID.21214954	Median signature	PMID.21214954
HS_Red4_BMC.Med.Genomics.2011_PMID.21214954	Median signature	PMID.21214954
Unknown_11_BMC.Med.Genomics.2011_PMID.21214954	Median signature	PMID.21214954
15q25_Amplicon_BMC.Med.Genomics.2011_PMID.21214954	Median signature	PMID.21214954
HS_Red14_BMC.Med.Genomics.2011_PMID.21214954	Median signature	PMID.21214954
MK14_K17_BMC.Med.Genomics.2011_PMID.21214954	Median signature	PMID.21214954
GSEA_GP19_1Q_amplicon.r=0.967_PerouLab_HS_Green17_ BMC_Med_Genomics_2011_PMID.21214954	Median signature	PMID.21214954
HS_Red22_BMC.Med.Genomics.2011_PMID.21214954	Median signature	PMID.21214954
HS_Green20_BMC.Med.Genomics.2011_PMID.21214954	Median signature	PMID.21214954
HS_Green22_BMC.Med.Genomics.2011_PMID.21214954	Median signature	PMID.21214954
MProtocadherin_BMC.Med.Genomics.2011_PMID.21214954	Median signature	PMID.21214954
HS_Green8_BMC.Med.Genomics.2011_PMID.21214954	Median signature	PMID.21214954
HS_Red19_BMC.Med.Genomics.2011_PMID.21214954	Median signature	PMID.21214954
HS_Red25_BMC.Med.Genomics.2011_PMID.21214954	Median signature	PMID.21214954
HS_Green18_BMC.Med.Genomics.2011_PMID.21214954	Median signature	PMID.21214954
HS_Green24_BMC.Med.Genomics.2011_PMID.21214954	Median signature	PMID.21214954
HS_Green13_BMC.Med.Genomics.2011_PMID.21214954	Median signature	PMID.21214954
HS_Green9_BMC.Med.Genomics.2011_PMID.21214954	Median signature	PMID.21214954
HS_Red15_BMC.Med.Genomics.2011_PMID.21214954	Median signature	PMID.21214954
HS_Green14_BMC.Med.Genomics.2011_PMID.21214954	Median signature	PMID.21214954
MITO2_BMC.Med.Genomics.2011_PMID.21214954	Median signature	PMID.21214954
HS_Red21_BMC.Med.Genomics.2011_PMID.21214954	Median signature	PMID.21214954
HS_Red20_BMC.Med.Genomics.2011_PMID.21214954	Median signature	PMID.21214954

**Signature_ID_paper**	**Signature type**	**PMID**
HS_Red24_BMC.Med.Genomics.2011_PMID.21214954	Median signature	PMID.21214954
HS_Green25_BMC.Med.Genomics.2011_PMID.21214954	Median signature	PMID.21214954
Fibroblast_Cluster_BMC.Med.Genomics.2011_PMID.21214954	Median signature	PMID.21214954
HS_Green19_BMC.Med.Genomics.2011_PMID.21214954	Median signature	PMID.21214954
MM_Green12_BMC.Med.Genomics.2011_PMID.21214954	Median signature	PMID.21214954
MM_Red17_BMC.Med.Genomics.2011_PMID.21214954	Median signature	PMID.21214954
MSquamous_BMC.Med.Genomics.2011_PMID.21214954	Median signature	PMID.21214954
MM_Red13_BMC.Med.Genomics.2011_PMID.21214954	Median signature	PMID.21214954
3p21Amplicon_BMC.Med.Genomics.2011_PMID.21214954	Median signature	PMID.21214954
MECM_BMC.Med.Genomics.2011_PMID.21214954	Median signature	PMID.21214954
Unknown_16_BMC.Med.Genomics.2011_PMID.21214954	Median signature	PMID.21214954
MNB2_BMC.Med.Genomics.2011_PMID.21214954	Median signature	PMID.21214954
GSEA_GP22_16Q22_24_amplicon.r=0.927_PerouLab_16q24x_<br>BMC_Med_Genomics_2011_PMID.21214954	Median signature	PMID.21214954
MNB3_BMC.Med.Genomics.2011_PMID.21214954	Median signature	PMID.21214954
Unknown_8_BMC.Med.Genomics.2011_PMID.21214954	Median signature	PMID.21214954
MBASAL_BMC.Med.Genomics.2011_PMID.21214954	Median signature	PMID.21214954
Unknown_7_BMC.Med.Genomics.2011_PMID.21214954	Median signature	PMID.21214954
Proliferation_Cluster_BMC.Med.Genomics.2011_PMID.21214954	Median signature	PMID.21214954
1p36_Amplicon_BMC.Med.Genomics.2011_PMID.21214954	Median signature	PMID.21214954
Unknown_14_BMC.Med.Genomics.2011_PMID.21214954	Median signature	PMID.21214954
MProliferation_BMC.Med.Genomics.2011_PMID.21214954	Median signature	PMID.21214954
16q23_Amplicon_BMC.Med.Genomics.2011_PMID.21214954	Median signature	PMID.21214954
11q13_Amplicon_BMC.Med.Genomics.2011_PMID.21214954	Median signature	PMID.21214954
S100A9_A8_BMC.Med.Genomics.2011_PMID.21214954	Median signature	PMID.21214954

**Signature_ID_paper**	**Signature type**	**PMID**
8p22_Amplicon_BMC.Med.Genomics.2011_PMID.21214954	Median signature	PMID.21214954
13q14_Amplicon_BMC.Med.Genomics.2011_PMID.21214954	Median signature	PMID.21214954
12qMDM4_BMC.Med.Genomics.2011_PMID.21214954	Median signature	PMID.21214954
MNB1_BMC.Med.Genomics.2011_PMID.21214954	Median signature	PMID.21214954
LUMINAL_Cluster_BMC.Med.Genomics.2011_PMID.21214954	Median signature	PMID.21214954
MM_Red23_BMC.Med.Genomics.2011_PMID.21214954	Median signature	PMID.21214954
ADM_S100A10_A110NDGR1_Cluster_ BMC.Med.Genomics.2011_PMID.21214954	Median signature	PMID.21214954
Unknown_10_BMC.Med.Genomics.2011_PMID.21214954	Median signature	PMID.21214954
Unknown_12_BMC.Med.Genomics.2011_PMID.21214954	Median signature	PMID.21214954
MFGFR2_BMC.Med.Genomics.2011_PMID.21214954	Median signature	PMID.21214954
MM_Green19_BMC.Med.Genomics.2011_PMID.21214954	Median signature	PMID.21214954
17q25x_BMC.Med.Genomics.2011_PMID.21214954	Median signature	PMID.21214954
MNOtch4_BMC.Med.Genomics.2011_PMID.21214954	Median signature	PMID.21214954
Unknown_9_BMC.Med.Genomics.2011_PMID.21214954	Median signature	PMID.21214954
MNADH_CYTochrome_BMC.Med.Genomics.2011_PMID.21214954	Median signature	PMID.21214954
MDACC_P53_ER.Pos_CCR.2011_PMID.21248301	Median signature	PMID.21248301
Early_Response_ER.Neg_27_JAMA.2011_PMID.21558518	Median signature	PMID.21558518
Excellent_Pathologic_Response_ER.Pos_39_JAMA.2011_PMID.21558518	Median signature	PMID.21558518
Excellent_Pathologic_Response_ER.Neg_55_JAMA.2011_PMID.21558518	Median signature	PMID.21558518
Early_Relapse_ER.Pos_33_JAMA.2011_PMID.21558518	Median signature	PMID.21558518
Extensive_Residual_Diesase_ER.Neg_54_JAMA.2011_PMID.21558518	Median signature	PMID.21558518
Extensive_Residual_Diesase_ER.Pos_73_JAMA.2011_PMID.21558518	Median signature	PMID.21558518
TNBC_Clinically_Relevant_Good.26_BCR.2011_PMID.21978456	Median signature	PMID.21978456
TNBC_Clinically_Relevant_Poor.26_BCR.2011_PMID.21978456	Median signature	PMID.21978456

Signature_ID_paper	Signature type	PMID
TNBC_Clinically_Relevant_Good.230_BCR.2011_PMID.21978456	Median signature	PMID.21978456
5Q_Breast.Cancer.Res.Treat.2012_PMID.22048815	Median signature	PMID.22048815
Wahl_aMaSC_Signature_Cell.Stem.Cell.2012_PMID.22305568	Median signature	PMID.22305568
Wahl_fMasC_Signature_Cell.Stem.Cell.2012_PMID.22305568	Median signature	PMID.22305568
Wahl_fSTR_Signature_Cell.Stem.Cell.2012_PMID.22305568	Median signature	PMID.22305568
PIK3CA.Hutti_Cancer.Res.2012_PMID.22552288	Median signature	PMID.22552288
Response_to_PARP_inhibitor_olaparib_Resistance_ BC_Res_Treat_2012_PMID.22875744	Median signature	PMID.22875744
Response_to_PARP_inhibitor_olaparib_Sensitivity_ BC_Res_Treat_2012_PMID.22875744	Median signature	PMID.22875744
Inflammatory_Breast_Cancer_491_nIBC_CCR.2013_PMID.23396049	Median signature	PMID.23396049
Inflammatory_Breast_Cancer_79_IBC_CCR.2013_PMID.23396049	Median signature	PMID.23396049
Inflammatory_Breast_Cancer_491_IBC_CCR.2013_PMID.23396049	Median signature	PMID.23396049
Inflammatory_Breast_Cancer_79_nIBC_CCR.2013_PMID.23396049	Median signature	PMID.23396049
Miller_Proliferation_Metagene_Genome_Biol_2013_PMID.23618380	Median signature	PMID.23618380
C3_TAG_RESPONDING_Usary_Clin.Cancer.Res.2013_PMID.23780888	Median signature	PMID.23780888
C3_TAG_UNTREATED_Usary_Clin.Cancer.Res.2013_PMID.23780888	Median signature	PMID.23780888
Vascular.Content_Clin.Exp.Metastasis.2014_PMID.23975155	Median signature	PMID.23975155
Activate.Endothelium_Clin.Exp.Metastasis.2014_PMID.23975155	Median signature	PMID.23975155
HGF_up_BCR.2013_PMID.24025166	Median signature	PMID.24025166
HGF_down_BCR.2013_PMID.24025166	Median signature	PMID.24025166
Verhaak_Stromal_Nat.Commun.2013_PMID.24113773	Median signature	PMID.24113773
IMMUNE_Bindea_Cell_Normal_mucosa_Immunity.2013_PMID.24138885	Median signature	PMID.24138885
IMMUNE_Bindea_Cell_SW480_cancer_cells_Immunity.2013_PMID.24138885	Median signature	PMID.24138885
IMMUNE_Bindea_Cell_Lymph_vessels_Immunity.2013_PMID.24138885	Median signature	PMID.24138885
MM_p53null.Luminal_Genome.Biol.2013_PMID.24220145	Median signature	PMID.24220145

**Signature_ID_paper**	**Signature type**	**PMID**
MM_Neu.2012_Genome.Biol.2013_PMID.24220145	Median signature	PMID.24220145
MM_Erbb2.like_Genome.Biol.2013_PMID.24220145	Median signature	PMID.24220145
MM_Stat1_Genome.Biol.2013_PMID.24220145	Median signature	PMID.24220145
MM_Claudinlow_Genome.Biol.2013_PMID.24220145	Median signature	PMID.24220145
MM_Myoepithelioma.like_Genome.Biol.2013_PMID.24220145	Median signature	PMID.24220145
MM_Squamous.like_Genome.Biol.2013_PMID.24220145	Median signature	PMID.24220145
MM_p53null.Basal_Genome.Biol.2013_PMID.24220145	Median signature	PMID.24220145
MM_Wnt1.Early_Genome.Biol.2013_PMID.24220145	Median signature	PMID.24220145
MM_C3Tag.2012_Genome.Biol.2013_PMID.24220145	Median signature	PMID.24220145
MM_PyMT.2012_Genome.Biol.2013_PMID.24220145	Median signature	PMID.24220145
MM_Class8_Genome.Biol.2013_PMID.24220145	Median signature	PMID.24220145
MM_Wnt1.Late_Genome.Biol.2013_PMID.24220145	Median signature	PMID.24220145
MM_Class3_Genome.Biol.2013_PMID.24220145	Median signature	PMID.24220145
Endothelial_Normal_EC_high_Angiogenesis_2014_PMID.24257808	Median signature	PMID.24257808
Endothelial_Tumor_EC_high_Angiogenesis_2014_PMID.24257808	Median signature	PMID.24257808
BPMS_7.Genes_PLoS.One.2013_PMID.24349199	Median signature	PMID.24349199
JANES_Oscillation_JUND_KRT5_Nat.Cell.Biol.2014_PMID.24658685	Median signature	PMID.24658685
JANES_Oscillation_GDF11_TGFBR3_Nat.Cell.Biol.2014_PMID.24658685	Median signature	PMID.24658685
XBP1_Signature_Nature.2014_PMID.24670641	Median signature	PMID.24670641
Parity_signature_Troester_40_BCR.2014_PMID.25005139	Median signature	PMID.25005139
Parity_signature_Troester_251_BCR.2014_PMID.25005139	Median signature	PMID.25005139
Knudsen_Neo_ER_negative_Clin.Cancer.Res.2014_PMID.25047707	Median signature	PMID.25047707
Knudsen_Neo_common_Clin.Cancer.Res.2014_PMID.25047707	Median signature	PMID.25047707
Knudsen_Neo_ER_positive_Clin.Cancer.Res.2014_PMID.25047707	Median signature	PMID.25047707
STAT3_Basal_Horvath_SHORT_PNAS.2014_PMID.25139989	Median signature	PMID.25139989

Signature_ID_paper	Signature type	PMID
STAT3_Basal_Horvath_PNAS.2014_PMID.25139989	Median signature	PMID.25139989
IntClust_Amplification.50_Genome.Biol.2014_PMID.25164602	Median signature	PMID.25164602
IntClust_Deletion.50.Better.than_Genome.Biol.2014_PMID.25164602	Median signature	PMID.25164602
IntClust_Amplification.50.Better.than_Genome.Biol.2014_PMID.25164602	Median signature	PMID.25164602
IntClust_Deletion.50_Genome.Biol.2014_PMID.25164602	Median signature	PMID.25164602
Stingl_Down_CLOW_High_Nat.Cell.Biol.2014_PMID.25173976	Median signature	PMID.25173976
Stingl_Day7_Downregulated_Nat.Cell.Biol.2014_PMID.25173976	Median signature	PMID.25173976
Stingl_Up_Basal_High_Nat.Cell.Biol.2014_PMID.25173976	Median signature	PMID.25173976
Stingl_Down_Basal_High_Nat.Cell.Biol.2014_PMID.25173976	Median signature	PMID.25173976
Stingl_Up_Proliferation_Nat.Cell.Biol.2014_PMID.25173976	Median signature	PMID.25173976
Stingl_Day7_Upregulated_Nat.Cell.Biol.2014_PMID.25173976	Median signature	PMID.25173976
MICRORNA_138_Signature_Cancer.Res.2014_PMID.25339353	Median signature	PMID.25339353
CD103_Positive_Cancer.Cell.2014_PMID.25446897	Median signature	PMID.25446897
aMaSC_HsEnriched_Refined1_BCR.2015_PMID.25575446	Median signature	PMID.25575446
Lim2010_MaSC_Adam_PMID.25575446	Median signature	PMID.25575446
Lim2009_MatureLum_Adam_PMID.25575446	Median signature	PMID.25575446
Lim2010_MatureLum_Adam_PMID.25575446	Median signature	PMID.25575446
Spike2012_fStr_PMID.25575446	Median signature	PMID.25575446
aMaSC_Shehata_BCR.2015_PMID.25575446	Median signature	PMID.25575446
Shehata2012_Basal_PMID.25575446	Median signature	PMID.25575446
aStr_Prat_BCR.2015_PMID.25575446	Median signature	PMID.25575446
Pfefferle2012_Stroma_PMID.25575446	Median signature	PMID.25575446
aStr_Shehata_BCR.2015_PMID.25575446	Median signature	PMID.25575446
Shehata2012_Stroma_PMID.25575446	Median signature	PMID.25575446
fMaSC.refined1_PMID.25575446	Median signature	PMID.25575446

**Signature_ID_paper**	**Signature type**	**PMID**
aMaSC_Lim09_BCR.2015_PMID.25575446	Median signature	PMID.25575446
Lim2009_MaSC_Adam_PMID.25575446	Median signature	PMID.25575446
Lim2010_LumProg_Adam_PMID.25575446	Median signature	PMID.25575446
Keller2012_CD10_Adam_PMID.25575446	Median signature	PMID.25575446
Lim2010_Stroma_Adam_PMID.25575446	Median signature	PMID.25575446
LumProg_Lim09_BCR.2015_PMID.25575446	Median signature	PMID.25575446
Lim2009_LumProg_Adam_PMID.25575446	Median signature	PMID.25575446
LumProg_Prat_BCR.2015_PMID.25575446	Median signature	PMID.25575446
Pfefferle2012_LumProg_PMID.25575446	Median signature	PMID.25575446
Shehata2012_ALDHneg_PMID.25575446	Median signature	PMID.25575446
Spike2012_fMaSC_PMID.25575446	Median signature	PMID.25575446
aStr_HsEnriched_Refined2_BCR.2015_PMID.25575446	Median signature	PMID.25575446
aMaSC_Prat_BCR.2015_PMID.25575446	Median signature	PMID.25575446
Pfefferle2012_MaSC_PMID.25575446	Median signature	PMID.25575446
aStr_HsEnriched_Refined1_BCR.2015_PMID.25575446	Median signature	PMID.25575446
aStr_Lim09_BCR.2015_PMID.25575446	Median signature	PMID.25575446
Lim2009_Stroma_Adam_PMID.25575446	Median signature	PMID.25575446
aStr_HsEnriched_BCR.2015_PMID.25575446	Median signature	PMID.25575446
LumProg_HsEnriched_Refined1_BCR.2015_PMID.25575446	Median signature	PMID.25575446
MatureLum_Prat_BCR.2015_PMID.25575446	Median signature	PMID.25575446
Pfefferle2012_MatureLum_PMID.25575446	Median signature	PMID.25575446
aMaSC_HsEnriched_BCR.2015_PMID.25575446	Median signature	PMID.25575446
MatureLum_HsEnriched_BCR.2015_PMID.25575446	Median signature	PMID.25575446
MatureLum_Shehata_BCR.2015_PMID.25575446	Median signature	PMID.25575446
Shehata2012_NCL_PMID.25575446	Median signature	PMID.25575446

**Signature_ID_paper**	**Signature type**	**PMID**
Spike2012_aMaSC_PMID.25575446	Median signature	PMID.25575446
LumProg_HsEnriched_BCR.2015_PMID.25575446	Median signature	PMID.25575446
Shehata2012_Erbb3neg_PMID.25575446	Median signature	PMID.25575446
MatureLum_Lim09_BCR.2015_PMID.25575446	Median signature	PMID.25575446
MatureLum_HsEnriched_Refined1_BCR.2015_PMID.25575446	Median signature	PMID.25575446
LumProg_Shehata_BCR.2015_PMID.25575446	Median signature	PMID.25575446
Shehata2012_ALDHpos_PMID.25575446	Median signature	PMID.25575446
Shehata2012_LumProg_PMID.25575446	Median signature	PMID.25575446
MIR_200c_Repressed_ONCO.2015_PMID.25746005	Median signature	PMID.25746005
MIR_200c_Induced_ONCO.2015_PMID.25746005	Median signature	PMID.25746005
Up_regulated_upon_N_RAS_repression_Cell_Rep.2015_PMID.26166574	Median signature	PMID.26166574
Up_regulated_by_Oncogenic_N_RAS_Cell_Rep.2015_PMID.26166574	Median signature	PMID.26166574
Down_regulated_upon_N_RAS_repression_Cell_Rep.2015_PMID.26166574	Median signature	PMID.26166574
AGE_associated_SOOD_Genome.Biol.2015_PMID.26343147	Median signature	PMID.26343147
GO_UP_with_SOX10_OE_Cell.Rep.2015_PMID.26365194	Median signature	PMID.26365194
GO_DOWN_with_SOX10_OE_Cell.Rep.2015_PMID.26365194	Median signature	PMID.26365194
TCGA.BRCA.1198_UNKNOWN6_Cell.2015_PMID.26451490	Median signature	PMID.26451490
TCGA.BRCA.1198_NORMAL_Cell.2015_PMID.26451490	Median signature	PMID.26451490
TCGA.BRCA.1198_UNKNOWN3_Cell.2015_PMID.26451490	Median signature	PMID.26451490
TCGA.BRCA.1198_COLLAGEN11A_Cell.2015_PMID.26451490	Median signature	PMID.26451490
TCGA.BRCA.1198_TP63_Cell.2015_PMID.26451490	Median signature	PMID.26451490
TCGA.BRCA.1198_LUMINAL_Cell.2015_PMID.26451490	Median signature	PMID.26451490
LOBULAR_TCGA_SIGNATURE_Reactive_like_Cell.2015_PMID.26451490	Median signature	PMID.26451490
TCGA.BRCA.1198_S100A7_8_9_Cell.2015_PMID.26451490	Median signature	PMID.26451490
LOBULAR_TCGA_SIGNATURE_Immune_related_Cell.2015_PMID.26451490	Median signature	PMID.26451490

**Signature_ID_paper**	**Signature type**	**PMID**
TCGA.BRCA.1198_IL8_CCL_Cell.2015_PMID.26451490	Median signature	PMID.26451490
TCGA.BRCA.1198_FGFR4_EGF_Cell.2015_PMID.26451490	Median signature	PMID.26451490
TCGA.BRCA.1198_HOXC11_HOTAIR_SIX1_Cell.2015_PMID.26451490	Median signature	PMID.26451490
TCGA.BRCA.1198_UNKNOWN2_Cell.2015_PMID.26451490	Median signature	PMID.26451490
TCGA.BRCA.1198_NORMAL2_Cell.2015_PMID.26451490	Median signature	PMID.26451490
TCGA.BRCA.1198_HISTONES_Cell.2015_PMID.26451490	Median signature	PMID.26451490
TCGA.BRCA.1198_Chromogranin_Cell.2015_PMID.26451490	Median signature	PMID.26451490
TCGA.BRCA.1198_BASAL_Cell.2015_PMID.26451490	Median signature	PMID.26451490
LOBULAR_TCGA_SUBTYPE_Proliferative_Cell.2015_PMID.26451490	Median signature	PMID.26451490
TCGA.BRCA.1198_PDCHA_MANY_Cell.2015_PMID.26451490	Median signature	PMID.26451490
TCGA.BRCA.1198_EN1_FDZ9_Cell.2015_PMID.26451490	Median signature	PMID.26451490
TCGA.BRCA.1198_UNKNOWN1_Cell.2015_PMID.26451490	Median signature	PMID.26451490
TCGA.BRCA.1198_MYBL2_APOBEC3B_Cell.2015_PMID.26451490	Median signature	PMID.26451490
LOBULAR_TCGA_SUBTYPE_Immune_related_Cell.2015_PMID.26451490	Median signature	PMID.26451490
TCGA.BRCA.1198_UNKNOWN5_Cell.2015_PMID.26451490	Median signature	PMID.26451490
LOBULAR_TCGA_SUBTYPE_Reactive_like_Cell.2015_PMID.26451490	Median signature	PMID.26451490
GSEA_GP21_Anti_apoptosis_DNA_stability.r=0.898_MORF_STK17A	Median signature	PMID.26771021
GSEA_CASPASE_CASCADE_APOPTOSIS_SA	Median signature	PMID.26771022
GSEA_GP21_Anti_apoptosis_DNA_stability.r=0.925_MORF_MT4	Median signature	PMID.26771023
GSEA_GP18_Vesicle_EPR_membrane_coat.r=0.877_MEMBRANE_COAT	Median signature	PMID.26771024
GSEA_BIOCARTA_RB_PATHWAY	Median signature	PMID.26771025
GSEA_RETINOL_METABOLISM_KEGG	Median signature	PMID.26771026
GSEA_GP3_Tumor_suppressing_miRNA_targets.r=0.940_GTTTGTT.MIR_495	Median signature	PMID.26771027
GSEA_GP12_Hypoxia_glycolosis.r=0.939_SEMENZA_HIF1_TARGETS	Median signature	PMID.26771028
GSEA_GP1_Proliferation_DNA_repair.r=0.981_PUJANA_CHEK2_PCC_NETWORK	Median signature	PMID.26771029

**Signature_ID_paper**	**Signature type**	**PMID**
GSEA_RESPONSE_TO_ANDROGEN_UP_NELSON	Median signature	PMID.26771030
GSEA_GP20_TAL1_Leukemia_erythropoiesis.r=0.935_GNF2_TAL1	Median signature	PMID.26771031
GSEA_BIOCARTA_RAS_PATHWAY	Median signature	PMID.26771032
GSEA_GP3_Tumor_suppressing_miRNA_targets.r=0.952_ DACOSTA_UV_RESPONSE_VIA_ERCC3_DN	Median signature	PMID.26771033
GSEA_GP8_FOXO_stemness.r=0.931_TTGTTT_V$FOXO4_01	Median signature	PMID.26771034
GSEA_MTOR_PATHWAY_BIOCARTA	Median signature	PMID.26771035
GSEA_BIOCARTA_VEGF_PATHWAY	Median signature	PMID.26771036
GSEA_BIOCARTA_PTEN_PATHWAY	Median signature	PMID.26771037
GSEA_GP10_Fatty_acid_oxidation.r=0.930_CARBOXYLIC_ACID_METABOLIC_PROCESS	Median signature	PMID.26771038
GSEA_IGF1R_PATHWAY_BIOCARTA	Median signature	PMID.26771039
GSEA_GP16_Protein_kinase_signailng_MAPKs.r=0.893_ INTRACELLULAR_SIGNALING_CASCADE	Median signature	PMID.26771040
GSEA_GP21_Anti_apoptosis_DNA_stability.r=0.877_MORF_BCL2	Median signature	PMID.26771041
GSEA_GP6_Squamous_differentiation_development.r=0.902_ RICKMAN_TUMOR_DIFFERENTIATED_WELL_VS_POORLY_DN	Median signature	PMID.26771042
GSEA_BIOCARTA_ALK_PATHWAY	Median signature	PMID.26771043
GSEA_GP14_Plasma_membrane_cell_cell_signaling.r=0.925_MORF_CNTN1	Median signature	PMID.26771044
GSEA_GP8_FOXO_stemness.r=0.875_MORF_PTPRB	Median signature	PMID.26771045
GSEA_BIOCARTA_AKT_PATHWAY	Median signature	PMID.26771046
GSEA_HDAC_TARGETS_DN_HELLER	Median signature	PMID.26771047
GSEA_GP1_Proliferation_DNA_repair.r=0.972_REACTOME_CELL_CYCLE_MITOTIC	Median signature	PMID.26771048
GSEA_GP7_Estrogen_signaling.r=0.97_SMID_BREAST_CANCER_BASAL_DN	Median signature	PMID.26771049
GSEA_BIOCARTA_BRCA_ATR_PATHWAY_ATRBRCA	Median signature	PMID.26771050
GSEA_GP13_Neural_signailng.r=0.959_MODULE_100	Median signature	PMID.26771051
GSEA_GP16_Protein_kinase_signailng_MAPKs.r=0.885_REGULATION_OF_KINASE_ACTIVITY	Median signature	PMID.26771052

**Signature_ID_paper**	**Signature type**	**PMID**
GSEA_GP17_Basal_signaling.r=0.958_SMID_BREAST_CANCER_BASAL_UP	Median signature	PMID.26771053
GSEA_GP3_Tumor_suppressing_miRNA_targets.r=0.940_TGCTTTG.MIR_330	Median signature	PMID.26771054
GSEA_GP13_Neural_signailng.r=0.894_NERVOUS_SYSTEM_DEVELOPMENT	Median signature	PMID.26771055
GSEA_PI3K_CASCADE_REACTOME	Median signature	PMID.26771056
GSEA_MYC_amplified_chr8q24	Median signature	PMID.26771057
FOXC1_Hair_Follicles_Wang_SCIENCE.2016_P30C.LO.vs.WT_Negative_PMID.26912704	Median signature	PMID.26912704
FOXC1_Hair_Follicles_Wang_SCIENCE.2016_P30C.LO.vs.WT_Positive_PMID.26912704	Median signature	PMID.26912704
Early_IRS_1_PLoS.One.2016_PMID.26991655	Median signature	PMID.26991655
Early_IRS_2_PLoS.One.2016_PMID.26991655	Median signature	PMID.26991655
Late_IRS_2_PLoS.One.2016_PMID.26991655	Median signature	PMID.26991655
Late_IRS_1_PLoS.One.2016_PMID.26991655	Median signature	PMID.26991655
Tumour_hypoxia_causes_DNA_hypermethylation_by_reducing_TET_activity_ Nature.2016_PMID.27533040	Median signature	PMID.27533040
Necrosis_J.Pathol.2017_PMID.27861902	Median signature	PMID.27861902
Mitotic_Count_J.Pathol.2017_PMID.27861902	Median signature	PMID.27861902
Apocrine_Features_J.Pathol.2017_PMID.27861902	Median signature	PMID.27861902
Histological_Grade_J.Pathol.2017_PMID.27861902	Median signature	PMID.27861902
Epithelial_Tubule_Formation_J.Pathol.2017_PMID.27861902	Median signature	PMID.27861902
Ductal_Carcinoma_In_Situ_J.Pathol.2017_PMID.27861902	Median signature	PMID.27861902
Stromal_Central_Fibrotic_Focus_J.Pathol.2017_PMID.27861902	Median signature	PMID.27861902
Nuclear_Pleomorphism_J.Pathol.2017_PMID.27861902	Median signature	PMID.27861902
Lobular_Carcinoma_In_Situ_J.Pathol.2017_PMID.27861902	Median signature	PMID.27861902
Lymphovascular_Invasion_J.Pathol.2017_PMID.27861902	Median signature	PMID.27861902
YALE_PIK3CA_Pathway_Ann.Oncol.2017_PMID.28177460	Median signature	PMID.28177460
YALE_RHOA_pathway_Ann.Oncol.2017_PMID.28177460	Median signature	PMID.28177460

**Signature_ID_paper**	**Signature type**	**PMID**
PR_Isoform_Ratio_Up_in_PRB_H_JNCI.2017_PMID.28376177	Median signature	PMID.28376177
PR_Isoform_Ratio_Up_in_PRA_H_JNCI.2017_PMID.28376177	Median signature	PMID.28376177
PARP_Sensitivity_Signature_POSITIVE_Sci.Adv.2017_PMID.28439535	Median signature	PMID.28439535
PARP_Sensitivity_Signature_NEGATIVE_Sci.Adv.2017_PMID.28439535	Median signature	PMID.28439535
African_and_European_Ancestry_in_TCGA_Positive_JAMA.Oncol.2017_PMID.28472234	Median signature	PMID.28472234
African_and_European_Ancestry_in_TCGA_Negative_JAMA.Oncol.2017_PMID.28472234	Median signature	PMID.28472234
PARP_sensitivity_MDACC_NPJ.Syst.Biol.Appl._2017_PMID.28649435	Median signature	PMID.28649435
GENE_PANEL_UNCSEQ_7.1_Oncologist.2018_PMID.29158372	Median signature	PMID.29158372
MET_DOWN_Significant_Genes_LOW_BASALS_1_Genes_JCI.2018_PMID.29480819	Median signature	PMID.29480819
MET_UP_RNA_Seq_Significant_Genes_JCI.2018_PMID.29480819	Median signature	PMID.29480819
MET_DOWN_Significant_Genes_LOW_BASALS_2_Genes_JCI.2018_PMID.29480819	Median signature	PMID.29480819
MET_DOWN_RNA_Seq_Significant_Genes_JCI.2018_PMID.29480819	Median signature	PMID.29480819
MET_UP_Significant_Genes_HIGH_BASALS_Genes_JCI.2018_PMID.29480819	Median signature	PMID.29480819
Durvalumab_signature_Higgs_Clin.Cancer.Res.2018_PMID.29716923	Median signature	PMID.29716923
REPLICATION_STRESS_DOWN_SET_Cell.Rep.2018_PMID.29768207	Median signature	PMID.29768207
REPLICATION_STRESS_UP_SET_Cell.Rep.2018_PMID.29768207	Median signature	PMID.29768207
MAPK_pathway_activation_Wagle_NPJ.Precis.Oncol.2018_PMID.29872725	Median signature	PMID.29872725
fMaSC_Metab8_CellRep.2018_PMID.30089273	Median signature	PMID.30089273
fMaSC_Metab_CellRep.2018_PMID.30089273	Median signature	PMID.30089273
fMaSC_Signature_CellRep.2018_PMID.30089273	Median signature	PMID.30089273
ACTIVATED_CANCER_CELL_SIGNATURE_Nat.Cell.Biol.2019_PMID.31263265	Median signature	PMID.31263265
ACTIVATED_LUNG_MSC_SIGNATURE_Nat.Cell.Biol.2019_PMID.31263265	Median signature	PMID.31263265
