# Supplementary material for: Cell-cycle inhibition and immune microenvironment in breast cancer treated with ribociclib and letrozole or chemotherapy
Source: NPJ Breast Cancer. 2024 Mar 6;10:20. doi: 10.1038/s41523-024-00625-7 (PMC10918094; doi:10.1038/s41523-024-00625-7)
Supplement: Supplementary file 2 — reporting-summary [file 41523_2024_625_MOESM2_ESM.pdf]

nature portfolio

Corresponding author(s): Aleix Prat

Last updated by author(s): Jan 14, 2024

Reporting Summary

Nature Portfolio wishes to improve the reproducibility of the work that we publish. This form provides structure for consistency and transparency in reporting. For further information on Nature Portfolio policies, see our Editorial Policies and the Editorial Policy Checklist.

Statistics

For all statistical analyses, confirm that the following items are present in the figure legend, table legend, main text, or Methods section.

n/a	Confirmed	
☐	☒	The exact sample size (*n*) for each experimental group/condition, given as a discrete number and unit of measurement
☐	☐	A statement on whether measurements were taken from distinct samples or whether the same sample was measured repeatedly
☐	☒	The statistical test(s) used AND whether they are one- or two-sided<br>*Only common tests should be described solely by name; describe more complex techniques in the Methods section.*
☐	☒	A description of all covariates tested
☐	☒	A description of any assumptions or corrections, such as tests of normality and adjustment for multiple comparisons
☐	☒	A full description of the statistical parameters including central tendency (e.g. means) or other basic estimates (e.g. regression coefficient) AND variation (e.g. standard deviation) or associated estimates of uncertainty (e.g. confidence intervals)
☐	☒	For null hypothesis testing, the test statistic (e.g. *F*, *t*, *r*) with confidence intervals, effect sizes, degrees of freedom and *P* value noted<br>*Give P values as exact values whenever suitable.*
☒	☐	For Bayesian analysis, information on the choice of priors and Markov chain Monte Carlo settings
☒	☐	For hierarchical and complex designs, identification of the appropriate level for tests and full reporting of outcomes
☐	☒	Estimates of effect sizes (e.g. Cohen's *d*, Pearson's *r*), indicating how they were calculated

Our web collection on statistics for biologists contains articles on many of the points above.

Software and code

Policy information about availability of computer code

Data collection	Genomic analysis determination were made available without associated clinical data
Data analysis	All analyses were performed with R version 4.2.2 and Microsoft Excel.

For manuscripts utilizing custom algorithms or software that are central to the research but not yet described in published literature, software must be made available to editors and reviewers. We strongly encourage code deposition in a community repository (e.g. GitHub). See the Nature Portfolio guidelines for submitting code & software for further information.

Data

Policy information about availability of data

All manuscripts must include a data availability statement. This statement should provide the following information, where applicable:

- Accession codes, unique identifiers, or web links for publicly available datasets
- A description of any restrictions on data availability
- For clinical datasets or third party data, please ensure that the statement adheres to our policy

All datasets used and/or analyzed during the current study are available from the corresponding author on reasonable request.

Research involving human participants, their data, or biological material

Policy information about studies with human participants or human data. See also policy information about sex, gender (identity/presentation), and sexual orientation and race, ethnicity and racism.

Reporting on sex and gender	Eligible patients were women (≥18 years) who were postmenopausal. Neither men nor postmenopausal women were included in this study since LHRH agonist drugs were not contemplated.
Reporting on race, ethnicity, or other socially relevant groupings	Race and ethnicity were determined by the researchers and collected through the trial's eCRF.
Population characteristics	Eligible patients were women (≥18 years) who were postmenopausal with histologically confirmed invasive hormone-sensitive breast cancer, defined locally as hormone receptorpositive, HER2-negative by American Society of Clinical Oncology and College of American Pathologists guidelines, and centrally as luminal B by the standardised PAM50 (Prosigna) assay.7 Patients had operable stage I-IIIA breast cancer, with primary tumour size of at least 2 cm in diameter as measured by MRI. Other main inclusion criteria were baseline Eastern Cooperative Oncology Group (ECOG) performance status of 0–1, suitable haematological counts, and adequate hepatic and renal function. Patients with stage IV disease, or multifocal or bilateral breast cancer were ineligible. Patients were also excluded if they had other malignancies, inadequate bone marrow, impaired liver function, cardiac disease or history of cardiac dysfunction, including QT interval 450 ms or more, and uncontrolled hypertension.
Recruitment	SOLTI-1402 CORALLEEN is an open-label, two arm, conducted in Spain at 21 trial centers, parallel-arm, multicentre, randomised, open-label, phase 2 trial. From July 27, 2017 to Dec 7, 2018, 198 patients were assessed for eligibility and 106 were finally enrolled.
Ethics oversight	The study was done in accordance with Good Clinical Practice guidelines and the World Medical Association Declaration of Helsinki. Patients provided written, informed consent. Approvals for the study protocol were obtained from an independent ethics committee (Vall d'Hebron University Hospital, Barcelona, Spain).

Note that full information on the approval of the study protocol must also be provided in the manuscript.

Field-specific reporting

Please select the one below that is the best fit for your research. If you are not sure, read the appropriate sections before making your selection.

☒ Life sciences ☐ Behavioural & social sciences ☐ Ecological, evolutionary & environmental sciences

For a reference copy of the document with all sections, see nature.com/documents/nr-reporting-summary-flat.pdf

Life sciences study design

All studies must disclose on these points even when the disclosure is negative.

Sample size	Assuming that 20·0–25·0% of the patients in each group would reach low-ROR disease, the study would require a sample size of 47 patients per group for estimating the expected proportion with a 95% CI of plus or minus 11·5–12·4%. Assuming a proportion of patients of 10% dropped out, a total of 104 patients was planned to be recruited. Each group was designed to be analysed independently and the trial was not powered for formal comparison between groups.
Data exclusions	For RNAseq with more than 70% missing data were excluded. IN all other cases patients were excluded only if data were missing.
Replication	No replications were undertaken
Randomization	A total of 106 eligible patients were randomized. Eligible patients were randomly assigned (1:1) using a secure web-based system to ribociclib plus letrozole or multiagent chemotherapy. Randomisation was stratified by tumour size (T1–T2 vs T3) and nodal involvement using permuted blocks of 25 and an internet-based tool used to generate a pseudorandom number. Investigators enrolled participants and used identifying information to register them in the interactive web-response system. Randomisation was concealed, but neither participants nor investigators were masked to group assignment. All outcomes assessors were masked to clinical data.
Blinding	The study was not blinded, since it was open label, randomized, parallel, non-comparative phase II clinical trial

Reporting for specific materials, systems and methods

We require information from authors about some types of materials, experimental systems and methods used in many studies. Here, indicate whether each material, system or method listed is relevant to your study. If you are not sure if a list item applies to your research, read the appropriate section before selecting a response.

Materials & experimental systems

n/a	Involved in the study
☐	☒ Antibodies
☒	☐ Eukaryotic cell lines
☒	☐ Palaeontology and archaeology
☒	☐ Animals and other organisms
☐	☒ Clinical data
☒	☐ Dual use research of concern
☒	☐ Plants

Methods

n/a	Involved in the study
☒	☐ ChIP-seq
☒	☐ Flow cytometry
☒	☐ MRI-based neuroimaging

Antibodies

Antibodies used
An immunohistochemical (IHC) study for Ki67 was carried out with a mouse monoclonal primary antibody (clone MIB-1) reactive in FFPE tissue sections using a peroxidase-labeled detection system, standard antigen retrieved protocols and an automated immunostainer (Dako OMNIS, Glostrup, Denmark).

Validation
https://www.agilent.com/en/product/immunohistochemistry/antibodies-controls/primary-antibodies/ki-67-antigen-(dako-omnis)-76239

Clinical data

Policy information about clinical studies

All manuscripts should comply with the ICMJE guidelines for publication of clinical research and a completed CONSORT checklist must be included with all submissions.

Clinical trial registration
This trial was registered at ClinicalTrials.gov, NCT03248427

Study protocol
The study protocol can be found in the appendix.
https://www.thelancet.com/journals/lanonc/article/PIIS1470-2045(19)30786-7/fulltext

Data collection
SOLTI-1402 CORALLEEN is an open-label, two arm, conducted in Spain at 21 trial centers, parallel-arm, multicentre, randomised, open-label, phase 2 trial. From July 27, 2017 to Dec 7, 2018, 198 patients were assessed for eligibility and 106 were finally enrolled

Outcomes
The demographics and primary clinical endpoint of the patients treated in CORALLEEN have been previously published (Prat et al. Lancet Oncol 2020; 21: 33–43). Here, we report an extensive analysis of a high-risk ER+/HER2- cohort to understand the effects of neoadjuvant ribociclib and letrozole or multi-agent chemotherapy on the tumor cell cycle and tumor microenvironment by analyzing samples before, during, and after 6 months of therapy in patients with newly diagnosed PAM50 Luminal B breast cancer who participated in the SOLTI-1402 CORALLEEN phase II randomized trial.

Plants

Seed stocks
n/a

Novel plant genotypes
n/a

Authentication
n/a
